# Profiling of advanced glycation end products uncovers abiotic stress-specific target proteins in Arabidopsis

**DOI:** 10.1093/jxb/ery389

**Published:** 2018-11-05

**Authors:** Amanda K Chaplin, Igor Chernukhin, Ulrike Bechtold

**Affiliations:** School of Biological Sciences, University of Essex, Wivenhoe Park, Colchester, UK

**Keywords:** Abiotic stress, advanced glycation end products (AGEs), chloroplast, diurnal, drought, photosynthesis, protein glycation

## Abstract

Non-enzymatic post-translational modifications of proteins can occur when the nucleophilic amino acid side chains of lysine and arginine encounter a reactive metabolite to form advanced glycation end products (AGEs). Glycation arises predominantly from the degradation of reducing sugars, and glycation has been observed during metabolic stress from glucose metabolism in both animals and plants. The implications of glycating proteins on plant proteins and biology has received little attention, and here we describe a robust assessment of global glycation profiles. We identified 112 glycated proteins that were common under a range of growth conditions and abiotic stress treatments, but also showed rosette age, diurnal, and drought stress-specific targets. Among 18 drought stress-specific glycation targets included several thioredoxin and thioredoxin-like proteins. *In vitro* glycation of two carbohydrate metabolism enzymes led either to a reduction or to a complete inhibition of activity, demonstrating the impact of glycation on protein function. Taken together, our results suggest that stress-specific glycation patterns of a small number of regulatory proteins may have a much broader impact on downstream target proteins that are, for example, associated with primary metabolism.

## Teaser

General base levels of chloroplast protein glycation were independent of rosette age or stress status. Yet under different abiotic stress treatments, a few highly specific changes in glycation of potential regulatory proteins were detected.

## Introduction

In higher organisms, post-translational modifications (PTMs) modulate proteomic diversity by creating chemical changes to proteins following translation. In plants, >300 different types of PTMs have been identified that regulate enzyme activity, localization, stability, and signal transduction (Jensen, 2004; Rocks *et al.*, 2005; Stulemeijer and Joosten, 2008; Hunter, 2009; Deribe *et al.*, 2010; Maeda *et al.*, 2010; Duan and Walther, 2015). Attachment of carbohydrates to proteins to create glycoproteins is the most structurally diverse type of PTM that can significantly alter protein conformation (Parodi, 2000; Slawson *et al.*, 2006). Glycoproteins are complex, and are widely believed to transfer biological information as part of complex signalling pathways (Gabius *et al.*, 2011; Solís *et al*., 2015). Glycoproteins are either formed in a controlled manner via enzymatic (*N*- and *O*-glycosylation) reactions or in a spontaneous random manner by non-enzymatic (glycation) reactions. Both glycosylation and glycation are biologically important but differ significantly in their roles and reactions.

Glycation is a complex network of parallel and sequential reactions which is initiated by the carbonyl group of a reducing sugar (e.g. glucose, fructose, or galactose) reacting with a free amino group, followed by rearrangements to form early glycation products such as fructosyl-lysine (FL) and other ketoamines (McCance *et al.*, 1993; Thornalley *et al.*, 1999; Zhang *et al.*, 2009; Younus and Anwar, 2016). Further rearrangements and oxidation reactions subsequently lead to a heterogeneous group called advanced glycation end products (AGEs) (Neglia *et al.*, 1983; Baynes *et al.*, 1989). Additionally, AGEs can also be formed directly and relatively rapidly via reactive carbonyl compounds such as glyoxal and methylglyoxal (MG) (Thornalley *et al.*, 1999).

Due to their stable nature, AGEs can diffuse within an organism and propagate free radical reactions far from the site of formation, causing further damage to proteins, lipids, or DNA, acting as cytotoxic messengers (Hulbert *et al.*, 2007). Thus, AGEs are the primary cause for diabetic-, ageing-, chronic inflammation-, neurodegenerative-, and cancer-associated complications (Cerami, 1985; Brownlee *et al.*, 1988; McCance *et al.*, 1993; Tessier, 2010; Younus and Anwar, 2016). Despite their toxic nature, there is evidence that breakdown products of AGEs also have essential roles in signalling cascades by switching on defence pathways through the binding of AGE-containing peptides to receptors of AGEs (RAGEs) (Park *et al.*, 2010; Ott *et al.*, 2014). Binding of AGE-containing peptides initiates pro-inflammatory responses, activating NADPH oxidase, the production of reactive oxygen species (ROS), such as hydrogen peroxide (H_2_O_2_), and mitogen-activated protein kinase signalling pathways, inducing gene expression (Lander *et al.*, 1997; Dower and Qwarnstrom, 2003; Wautier and Schmidt, 2004; Alikhani *et al.*, 2007*a*, *b*).

MG is an important glycating agent and, despite there being detailed analyses on its production and detoxification via the glyoxalase pathway, analysis with respect to its damaging effects to proteins in plants has received little attention (Norton *et al.*, 1990; Maiti *et al.*, 1997; Thornalley *et al.*, 1999; Bechtold *et al.*, 2009). However, the development of proteomic tools and methods for simultaneous detection of glycation products has now made the analysis of products possible (Ahmed *et al.*, 2002; Thornalley *et al.*, 2003). For example, early stage FL and nine AGE-modified amino acids were identified in *Arabidopsis thaliana* (Arabidopsis) leaves under controlled conditions (Bechtold *et al.*, 2009). The physiological relevance of the occurrence of these products in Arabidopsis remains to be validated especially in relation to stress signalling. Nevertheless, it is well known that glucose levels in plants undergo diurnal variation, with the production of starch occurring during the light phase and starch degradation and release of glucose for respiration occurring during the dark phase. Elevations in mainly lysine-associated glycation products were observed during the dark period (Bechtold *et al.*, 2009), which coincides with an increase in H_2_O_2_ production (Bechtold *et al.*, 2004). Recent studies in *Brassica napus* and Arabidopsis identified 772 and 502 AGE-modified proteins in non-stressed leaf extracts, respectively (Bilova *et al.*, 2016), and identified osmotic stress-specific (Paudel *et al.*, 2016) and plant age-specific protein targets (Bilova *et al.*, 2017) in Arabidopsis. The general purpose of these studies was to identify the pathways and mechanisms of glycation in plants, identifying likely glycation substrates and specific glycation hotspots in plants. However, methodologies used did not clearly distinguish between glycosylated and glycated proteins, which may have caused an overestimation of protein targets. In addition, Bilova *et al*. (2016, 2017) do not provide an exhaustive list of all identified protein targets, nor do selected protein targets match across the different studies, making it difficult to judge the general impact of glycation in plants.

In the present study, we compared glycation patterns in Arabidopsis in response to short-term stresses such as high light or heat, a slow developing drought stress (Ferguson *et al.*, 2018), and changes in glycation targets during a diurnal period (Bechtold *et al.*, 2009) on soil-grown plants. The aim was to determine baseline levels of glycation, and to identify stress- and diurnal-specific protein targets, which would allow us to link glycation and protein function to underlying physiological effects, especially under specific stress treatments.

## Materials and methods

### Plant material, plant growth, and stress conditions

Arabidopsis ecotype Col-0 was obtained from the European Arabidopsis Stock Centre and seeds were planted in soil (Scotts Levington’s F2+S) as described in Ferguson *et al.* (2018). Following germination, seedlings were grown under a 8 h to 16 h light to dark cycle at 23 °C, 60% relative humidity, and light intensity of 150 µmol m^−2^ s^−1^ before being transferred into separate pots after 2 weeks. For heat and light stress experiments, plants were kept under constant well-watered conditions for 4 weeks before transferring plants into either high light [IsoLight at 850 µmol m^−2^ s^−1^ (Technologica)] or heat [37 °C, 78% humidity, vapour pressure deficit 1 kPa (Fitotron)] conditions for 4 h.

For drought conditions, all pots were filled with an identical weight of soil mix in order to determine relative soil water content (rSWC) as described in Ferguson *et al.* (2018). Drought stress was performed on 5-week-old plants, and material was harvested at ~40% and ~20% rSWC including well-watered controls. Plant materials were obtained from two independent experiments with six replicates for each stress treatment experiment.

### Chlorophyll fluorescence imaging

Plants were confirmed as responding to the heat or light stress conditions by dark adapting the plants before measuring the Chl *a* fluorescence parameter *F*_v_/*F*_m_ using a fluorescence imager (Fluorimager, Technologica, Colchester UK).

### Determination of glyoxalase II activity

Protein isolation and determination of glyoxalase II activity were carried out according to Bilova *et al.* (2016). For glyoxalase II activity, 50 μl of protein extracts were added to a mixture of 0.2 mM 5,5'-dithio-bis(2-nitrobenzoic acid (DTNB) and 1 mM *S*-d-lactoylglutathione in 20 mM NaH_2_PO_4_/Na_2_HPO_4_, pH 7.0 (450 μl). The absorbance at 412 nm was measured over 3–5 min (until no further change) with 30 s intervals using a spectrophotometer, and the activity was calculated using ε412 nm DTNB of 13 600 M^–1^ cm^–1^ (TNB^2–^ ion has a yellow colour and absorbance at 412 nm) (Ellman, 1959).

### Lipid hydroperoxides and H_2_O_2_ quantification

Lipid hydroperoxides (LHPOs) were quantified as described by Griffiths *et al.* (1997, 2000). Concentrations of LHPOs were determined using a 13(*S*)-hydroperoxy-9,11 (*cis, trans*)-octadecadienoic acid (13*S*-HPODE) calibration curve as standard. HPODE was prepared as described by Reeder and Wilson (1998) and calibration curves were constructed ranging from 1 μM to 150 μM.

H_2_O_2_ concentrations were determined by homogenizing 0.1 g (FW) of leaf tissue in 1 ml of 0.1 M HCl. Samples were centrifuged at 13000 rpm, 4 °C for 10 min, and the supernatant was cleared using activated charcoal. A 400 µl aliquot was then incubated with 2.6 ml of reaction mixture of 2.54 ml of 50 mM HEPES pH 7.5, 60 µl of 50 mM homovanillic acid, and 60 µl of 4 µM horseradish peroxidase (HRP; Sigma). Fluorescence was then measured at 425 nm emission (excitation of 315 nm) using an LS 50B fluorimeter (Perkin Elmer). H_2_O_2_ concentrations were determined using a H_2_O_2_ standard curve from 0.1 nmol to 10 nmol.

### Carbohydrate analysis

Soluble carbohydrates (glucose, fructose, and sucrose) and starch were extracted from 20 mg FW of leaf material. Extracts were incubated in 80% (v/v) ethanol for 30 min at 80 °C, then washed four times with 80% (v/v) ethanol. Glucose, fructose, and sucrose were measured from the ethanol extract using an enzyme-based protocol according to Stitt and Quick (1989). The starch content was estimated from the ethanol-insoluble pellet according to Stitt *et al.* (1978).

### Protein glycation

BSA, glyceraldehyde-3-phosphate dehydrogenase (GAPC1), and triosephosphate isomerase (TPI) were glycated according to Schmidt *et al.* (1992). BSA, GAPC1, and TPI were prepared with and without 0.4 M glucose and incubated at 37 °C in the dark for 3 weeks. Solutions were made using purified water, filtered prior to incubation, and contained various protease inhibitors to ensure minimal protein degradation and sterility during glycation. Solutions were then concentrated at 4 °C using a 5 kDa cut-off Centricon (Vivaspin) against 0.1 M sodium phosphate pH 7 buffer with two changes to remove any unspecific binding.

### Protein isolation, Rubisco fractionation, and alkaline hydrolysis

Proteins were isolated from three biological replicates. Approximately 1 g (FW) of plant material was homogenized and extracted in 6 ml of extraction buffer [20 mM MgCl_2_, 2% β-mercaptoethanol, 0.1% protease inhibitor cocktail, 1 mM phenylmethylsulphonyl fluoride (PMSF), 2% NP-40, 500 mM Tris-HCl, pH 8.3]. Extracts were cleared by filtration through a 0.2 µm Minisart filter (Sartorius) and centrifuged at 3000 *g*, 4 °C for 20 min. Rubisco was isolated using a 50% polyethylene glycol (PEG) 6000 solution adapted from Widjaja *et al.* (2009), producing an almost Rubisco-free supernatant and a Rubisco PEG fraction.

Proteins were solubilized in 250 μl of solubilization buffer (SB; 7 M urea, 2 M thiourea, 50 mM DTT, 4% CHAPS, 0.4% SDS, 5 mM K_2_CO_3_). For alkaline hydrolysis, an equal volume (250 μl) of 0.1 M NaOH (pH 13) was added and samples were incubated at 45 °C for 6 h. Reactions were then neutralized by addition of 250 μl of 0.1 M HCl.

### Boronate affinity chromatography (BAC)

Samples were adjusted to a volume of 2 ml with buffer A (250 mM ammonium acetate, 50 mM MgCl_2_, pH 8.1). Supernatants were loaded onto a 2 ml or 10 ml *m*-aminophenylboronic acid–agarose high performance Tricorn 5/100 column (GE healthcare) equilibrated with buffer A. Unbound non-glycated proteins were eluted in a 10 ml wash step with buffer A, before glycated proteins were eluted in 10 ml of 100% 0.1 M acetic acid using an ÄKTA pure purification system (GE Healthcare). Glycated fractions were collected and proteins were precipitated with acetone (4:1 acetone:water) overnight at –20 °C.

### Mass spectrometry

Precipitated proteins were resuspended in ~50 μl of SB and 10 μl of SDS loading dye, and heated at 95 °C for 3 min. Samples were then loaded onto a 12.5% SDS gel. Samples were allowed to enter the resolving portion of the gel before cutting out the gel slices and placing them in low bind siliconized tubes (Greiner). Gel slices were subjected to in-gel trypsin digestion as previously described (Metodieva *et al.*, 2013), and evaporated peptides were kept at –80 °C until required. The constituent tryptic peptides were concentrated and analysed by electrospray-ionization tandem MS on a hybrid high resolution LTQ/Orbitrap Velos instrument (Thermo Scientific, Hemel Hempstead, UK) interfaced to a split-less nanoscale HPLC (Ultimate 3000; Dionex, Loughborough, UK) as described previously (Greenwood *et al.*, 2012; Metodieva *et al.*, 2013; Croner *et al.*, 2014).

MaxQuant was used to analyse the MS/MS data (Cox *et al.*, 2014). Searches for the glycation sites were performed using MaxQuant Andromeda with masses of +58 Da for *N*-carboxymethyllysine/*N*-carboxymethylarginine (CML/CMA), +162 Da for FL, +39.99 Da for *N*-(5-hydro-4-imidazolon-2-yl)ornithine (G-H1), and +54 Da for *N*-(5-hydro-5-methyl-4-imidazolon-2-yl)ornithine (MG-H1).

### Data analysis

All statistical analysis was performed using R 3.4.3. The data from the 10%, SN, and 20% fractions were combined to cover the total glycated proteome for each condition. The DEP 1.1.4 package (Zhang *et al.*, 2018) was used to determine differential enrichment of glycated proteins between treatments. This package provides data filtering, variance normalization, imputation of missing values, and statistical testing. Proteins were filtered according to the presence in the biological replicates of, (i) a minimum of two out of three biological replicates (high stringency) or (ii) a minimum of one out of three of the biological replicates (low stringency) in at least one of the conditions tested.

One-way ANOVA and a post-hoc Tukey test was used to determine significance for the measurement of carbohydrates and stress biomarkers. Statistical significance for glycation targets was evaluated within the DEP 1.1.4 package (Zhang *et al.*, 2018). Values on the bar charts represent the mean ±SE (*n*=6) with the asterisk indicating significant differences at *P*<0.05.

### Western blotting

Protein solutions were quantified using the Bradford assay then diluted accordingly using SDS loading buffer. Samples were loaded on a 12.5% SDS–PAGE gel (BioRad) followed by transfer before incubation with the primary antibody (anti-CML at 1/1000 dilution, or anti-OGlcNAc at 1/5000). Goat anti-rabbit HRP secondary antibody added to anti-CML (1/2500, Promega), and goat anti-mouse HRP (1/5000, Thermo Fisher) added to anti-OGlcNAc were used to visualize the modifications using the Pierce ECL Western Blotting substrate (Thermo Scientific).

### Cloning, overexpression, and purification of GAPC1 and TPI

Genes encoding GAPC1 (accession no. P25858 encoding 338 amino acids) and TPI (accession no. Q9SKP6 encoding 254 amino acids) were cloned into pET28a expression vectors (BaseClear) and overexpressed in *Escherichia coli* BL21(DE3) cells. Recombinant proteins were purified once expressed at 37 °C in 1 litre of LB cultures after induction with 0.4 M isopropyl β-d-1-thiogalactopyranoside (IPTG; Melford). Cells were harvested 16 h after induction at 3501 *g* for 20 min at 4 °C. Pellets were resuspended in buffer C (50 mM Tris–HCl, 500 mM NaCl, 20 mM imidazole, pH 8) then lysed using an EmulsiFlex-C5 cell disrupter (Avestin) followed by centrifugation at 38724 *g* for 20 min at 4 °C. The clarified supernatant was then loaded onto a 5 ml Ni-NTA Sepharose column (GE Healthcare) and protein was eluted with a linear imidazole gradient using buffer D (buffer C with 500 mM imidazole) using an ÄKTA purification system. Single peaks were collected and concentrated at 4 °C using a Centricon (Vivaspin) with a 10 kDa cut-off. Concentrated protein was purified further using a G75 or S200 Superdex size exclusion column (GE Healthcare) equilibrated with buffer E (10 mM NaH_2_PO_4_/Na_2_HPO_4_, pH 7, 100 mM NaCl). Concentrations of purified protein were determined spectrophotometrically [Cary 60 UV-Visible spectrophotometer (Agilent)] using extinction coefficients (ε) of 40910 M^–1^ cm^-1^ and 38960 M^–1^ cm^–1^ for GAPC1 and TPI, respectively as determined using the ExPASy server (Gasteiger *et al.*, 2003).

### Steady-state enzyme activity

Steady-state enzyme kinetics were carried out with purified, non-glycated and glycated GAPC1 and TPI enzymes. The assays were measured using a Hewlett-Packard 8453 diode-array spectrophotometer scanning between 190 nm and 1100 nm at 25 °C. The GAPC1 assay was carried out in a 1 ml cuvette in 10 mM NaH_2_PO_4_/Na_2_HPO_4_, pH 8.5, 100 mM NaCl. Each reaction mixture contained 10 mM NAD^+^, 3 mM DTT, and 1 μM GAPC1 enzyme which was base-lined before the reaction was started by addition of 0–5 mM dl-glyeraldehyde-3-phosphate (G3P; Sigma). The TPI assay was also carried out in 1 ml cuvettes in 10 mM NaH_2_PO_4_/Na_2_HPO_4_, pH 7.0, 100 mM NaCl. Each reaction contained 0.1 mM NADH, 5 U of α-glycerophosphate dehydrogenase, and 0–5 mM G3P, and reactions were started by addition of 1 μM TPI enzyme. Plots of the turnover rate constant (*k*, s^–1^) versus substrate concentration were then constructed, whereby *k* is the initial rate normalized to the enzyme concentration calculated from {(Δ*A*/ε/*t*)/[enzyme]} where Δ*A* is the absorbance change at 340 nm (decrease for TPI with oxidation of NADH and increase for GAPC1 assay with reduction of NAD^+^), ε is the extinction coefficient of the substrate oxidation product which is 6.22 mM^–1^ cm^–1^, *t* is the time in seconds, and [enzyme] is the total concentration of the enzyme in the assay. The Michaelis constant, turnover number, and catalytic efficiency were estimated by non-linear least squares fitting to the Michaelis–Menten model.

## Results

### Specific enrichment for non-enzymatically glycated peptides

To distinguish between *N*-/*O*-glycosylation and non-enzymatic glycation (see the Introduction), glycated BSA was used to ensure that the enrichment was specific to non-enzymatic glycation modifications. Glycated BSA and the total leaf extract were separated via BAC into non-glycated/non-glycosylated and glycated/glycosylated peptide fractions (see Supplementary Fig. S1 at *JXB* online). Glycated BSA and leaf extracts were subsequently subjected to mild alkaline hydrolysis, neutralized, and separated again using a BAC column. Glycated BSA did not show a reduction in peak area (Supplementary Fig.S1A), owing to the fact that BSA only contained non-enzymatically glycated residues. However, in total leaf extracts the glycated/glycosylated peak was reduced by ~75% following alkaline hydrolysis (Supplementary Fig. S1B).

**Fig. 1.**
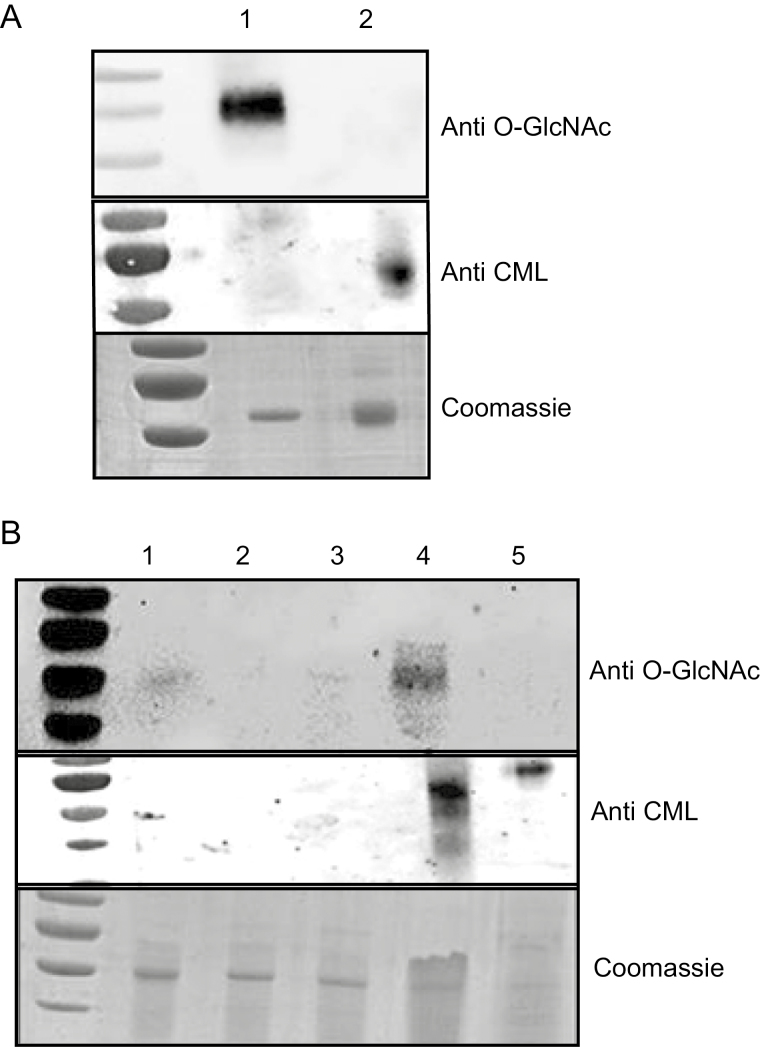
Enrichment for protein glycation after alkaline hydrolysis. (A) Specificity of anti-CML and anti-O-GlcNAc antibodies using modified BSA. Lane 1, *O*-glycosylated BSA (Thermo Fisher, UK); lane 2, non-enzymatically glycated BSA (see the Materials and methods). Top: a western blot hybridized with anti-O-GlcNAc. Middle: a western blot hybridized with anti-CML. Bottom: a Coomassie stain of an SDS–PAGE gel transferred onto a nitrocellulose membrane before western blot analysis. (B) Enrichment for glycated proteins before and after alkaline hydrolysis of total leaf extracts. Lane 1, total protein extract; lanes 2 and 3, non glycated peptide peaks; lane 4, glycated peak prior to alkaline hydrolysis; 5, glycated peptide peak post-alkaline hydrolysis. Top: a western blot hybridized with anti-O-GlcNAc. Middle: a western blot hybridized with anti-CML antibody. Bottom: a Coomassie stain of an SDS–PAGE gel before transfer to a nitrocellulose membrane for western blot analysis.

The separated glycated and non-glycated leaf fractions were subsequently analysed by western blot using glycation-specific anti-CML and *O*-glycosylation-specific anti-OGlcNAc antibodies. Specificity of the antibodies was verified using glycated and glycosylated BSA, respectively. The anti-CML antibody detected glycated BSA with minimal cross-reaction with *O*-glycosylated BSA (Fig. 1A), while anti-OGlcNAc exclusively identified *O*-glycosylated BSA (Fig. 1A). Importantly, anti-OGlcNAc and anti-CML both reacted with the leaf extract prior to alkaline hydrolysis (Fig. 1B), but following alkaline hydrolysis only the anti-CML reacted with the protein sample (Fig. 1B). Therefore, alkaline hydrolysis followed by BAC was successful in enriching for non-enzymatically glycated proteins, and illustrates that alkaline hydrolysis is essential in order to remove *N*- and *O*-glycosylated proteins.

### Stress treatments lead to increases in sugar levels and other stress biomarkers

**Fig. 2.**
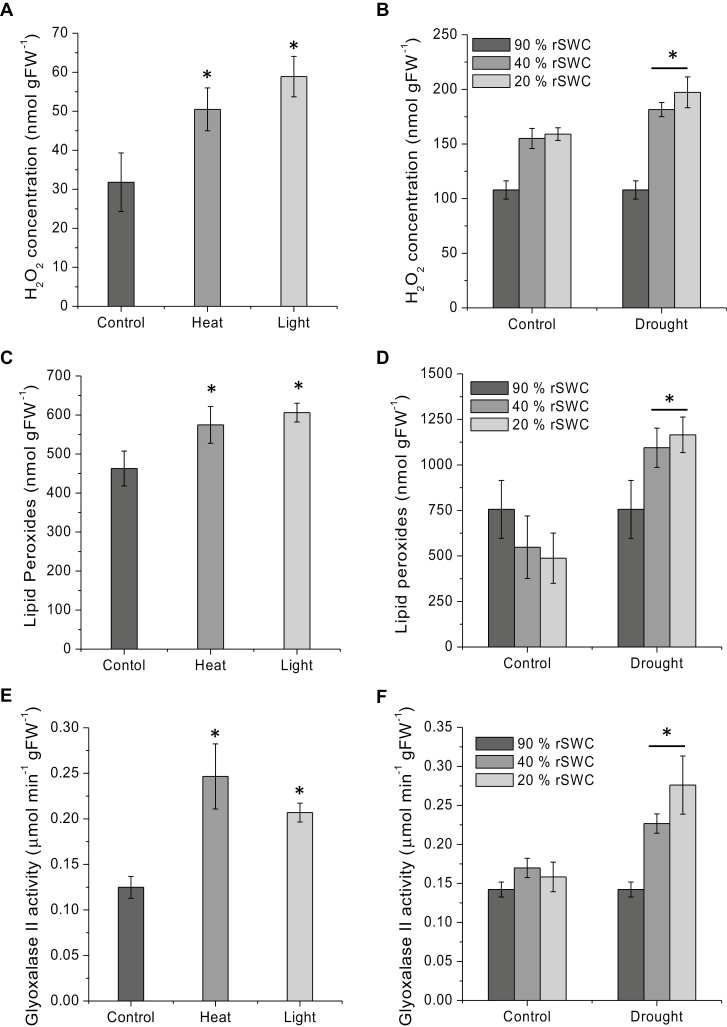
Analysis of stress response in Arabidopsis Col-0 plants under high light (IsoLight 850 µmol m^−2^ s^−1^), heat (37 °C, 78% humidity), and drought conditions. (A and B) H_2_O_2_ concentration (nmol gFW^–1^) measurements for (A) control, high light, and heat, and (B) drought stress treatments. H_2_O_2_ measurements were taken using homovanillic acid fluorescence by emission at 425 nm (excitation 315 nm) using an LS 50B fluorimeter. (C and D) Determination of lipid hydroperoxide content (nmol gFW^–1^) in (C) control, high light, and heat treatment, and (D) drought stress treatment. The concentrations were measured by ferrous oxidation-xylenol orange assay with absorption measurement at 560 nm using a Cary 60 UV-visible spectrophotometer. (Eand F) Glyoxalase II enzyme activity measurements of (E) control, high light, and heat, and (F) drought experiments. Measurements were taken with *S*-d-lactoylglutathione as substrate and the absorbance at 412 nm of DTNB was measured. * indicates a significant difference at *P*<0.05.

Arabidopsis rosettes were subjected to heat, light, and drought stress as described in Bechtold *et al.* (2009, 2013, 2016). To assess the physiological impact of heat or light stress, Chl *a* fluorescence imaging was used to measure the maximum quantum efficiency of PSII photochemistry (*F*_v_/*F*_m_). There was no significant difference between control and heat-stressed plants, with *F*_v_/*F*_m_ values of 0.8 ± 0.006 and 0.806 ± 0.007 for control and heat, respectively. Only in the high light treatment was a significant decrease in *F*_v_/*F*_m_ to 0.723 ± 0.006 observed (Supplementary Fig. S2). Heat, light, and drought stress treatments resulted in a significant increase in the production of H_2_O_2_ (Fig. 2A, B) and LHPOs (Fig. 2C, D). Glyoxalase II activity, a biomarker for increased glyoxal and methylglyoxal production, was also shown to increase significantly in either high light, heat, or drought stress treatments (Thornalley, 2003) (Fig. 2E, F). This suggests that the applied stress treatments were effective in inducing oxidative stress.

**Fig. 3.**
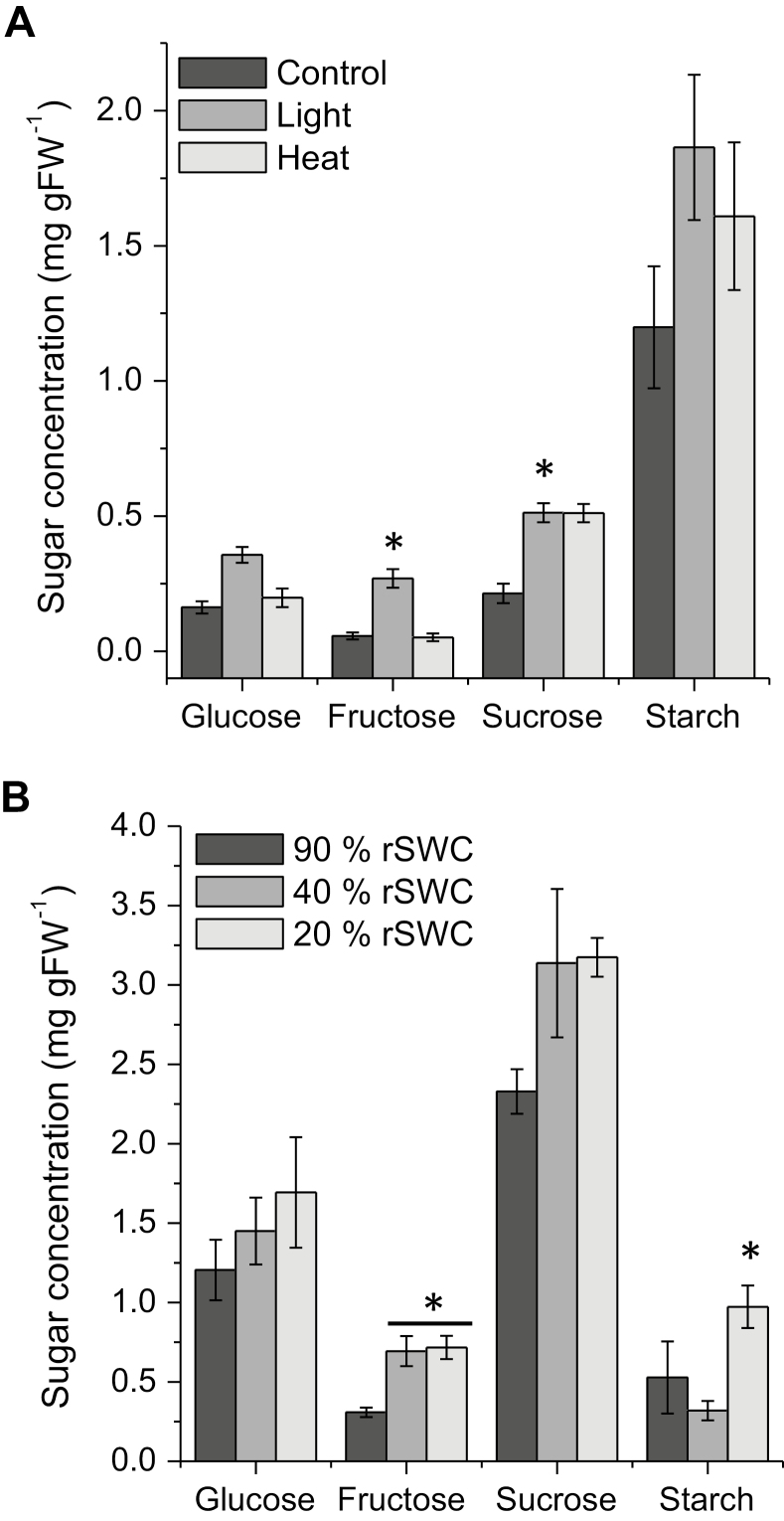
Carbohydrate analysis of high light, heat, and drought experiments in Arabidopsis. (A) Control, high light, and heat, and (B) drought analysis of soluble (glucose, fructose, and sucrose) and insoluble (starch) carbohydrates. * indicates a significant difference at *P*<0.05.

We also analysed glucose, fructose, sucrose, and starch content during stress treatments. High light or heat treatments led to significant increases in starch and sucrose content (Fig. 3A), while glucose and fructose only significantly increased during high light (Fig. 3A). Drought stress also significantly increased soluble carbohydrate levels but not the starch content (Fig. 3B). In addition, the expected diurnal changes in glucose, fructose, and sucrose were observed peaking during the light period, and decreasing during the night (Supplementary Fig. S3A).

### A core set of 112 proteins are targeted for glycation

**Fig. 4.**
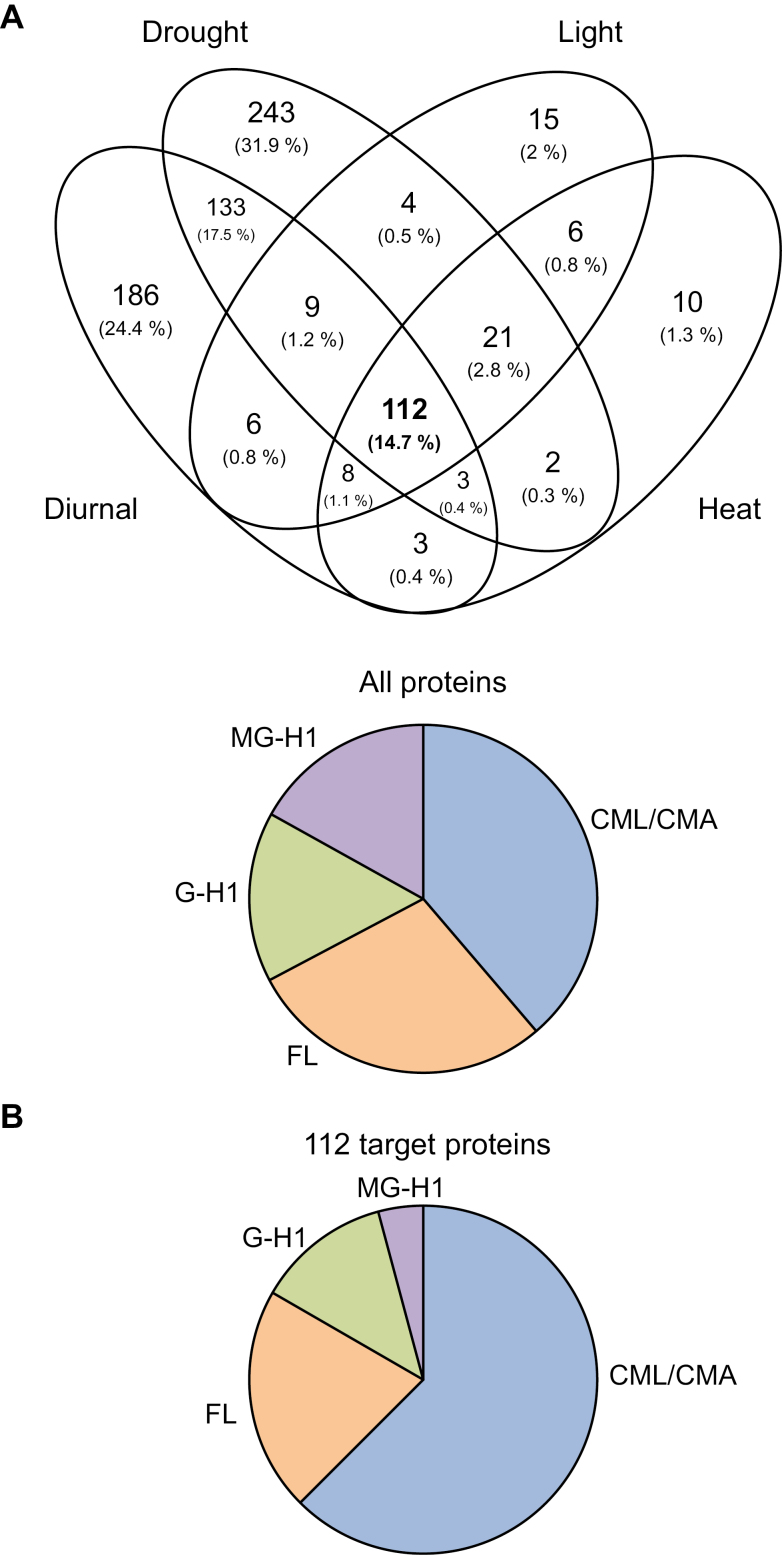
AGE-modified protein targets in Arabidopsis leaves. (A) Venn diagram showing the core 112 common proteins targeted for glycation identified in high light, heat, drought, and time-series experiments in Arabidopsis. The Venn diagram was constructed using Venny 2.1. (B) AGE-modified tryptic peptides obtained from Arabidopsis distributed by glycation modification in all the proteins in the high light, heat, and drought stress experiments, and in the 112 common target proteins. Searches for the glycation sites were performed using MaxQuant Andromeda with masses of +58 Da for *N*-carboxymethyllysine/*N*-carboxymethylarginine, +162 Da for fructosyl-lysine, +39.99 Da for *N*-(5-hydro-4-imidazolon-2-yl)ornithine, and +54 Da for *N*-(5-hydro-5-methyl-4-imidazolon-2-yl)ornithine.

Previous studies of glycation profiles have reported >500 protein targets in Arabidopsis leaf extracts; however, methodologies were unclear regarding the removal of glycosylated proteins, and may have resulted in an overestimation of targets (Bilova *et al.*, 2016). As described above, the use of alkaline hydrolysis removes the labile glycosylation and ribosylation moieties (Fig. 1; Supplementary Fig. S1), while leaving the stable covalently glycated peptides intact (Varki and Diaz, 1984). We identified 544, 182, 196, and 735 proteins during a diurnal period, heat, light, and drought experiments, respectively (see Supplementary Tables S1 and S2 for raw proteomics data). Generally, there were fewer targets in the heat and light experiments, which is probably due to plant age as high light and heat stress experiments were carried out on 4-week-old rosettes (Supplementray Table S3). These proteins contained thousands of peptides and 522, 502, 560, and 1863 glycation sites (modifications CML/CMA, FL, G-H1, and MG-H1) in the diurnal, heat, light, and drought experiments, respectively (Supplementary Table S3). An example mass spectrumof a CML glycated peptide belonging to the chloroplast-located PSBP1 oxygen-evolving enhancer protein 2-1 can be seen in Supplementary Fig. S4. All proteins were then subjected to a high stringency analysis, and any proteins with <2 unique peptides were removed, resulting in 459, 181, 165, and 527 proteins for a diurnal period, heat, light, and drought experiments, respectively. Comparison of all the proteins identified 112 common targets (Fig. 4A; Table 1). These 112 common targets were subsequently analysed using the DEP package (see the Materials and methods) (Zhang *et al.*, 2018). Some of the most abundant proteins amongst these targets include the β-subunit of the chloroplastic ATP synthase and chloroplastic phosphoglycerate kinase. Across all experiments, four main glycation modifications were searched for: CML/CMA, FL, G-H1, and MG-H1. The percentage weightings of the glycan modifications remained similar for all experiments. The glycation percentage proportions for heat, light, and drought experiments were similar (Fig. 4B). However, when the glycation sites were analysed in the 112 targets, significant differences in the proportions of glycation modification were evident. In all the proteins, 38.7% CMA/CML and 4.2% MG-H1 were identified, which both increased, to 62.5% and 16.9%, respectively, in the 112 common targets (Fig. 4B). Interestingly, very few of the 112 core targets (Table 1) significantly changed during the heat, light, and drought stress treatments (Table 2). The diurnal (08.00, 16.00, 24 h) and rosette age experiments (4 weeks, controls of the high light and heat experiment; 5 weeks, 90% rSWC control at the start of the drought experiment; and 6 weeks, 90% rSWC control at the end of the drought experiment), showed the majority of significant changes in the abundance of glycation targets. There were eight significant protein targets between 08.00 h and 24.00 h in the diurnal experiment, and 17 significant protein targets between 4- and 6-week-old rosettes (Table 2; Supplementary Fig. S5A).

**Table 1.** The112 common AGE-modified proteins identified in high light, heat, drought, and time-series experiments in Arabidopsis plants

Protein annotation		Genome identifier	Unique peptides
Protein name	Accession no.		
PROTOCHLOROPHYLLIDE OXIDOREDUCTASE C	O48741	AT1G03630	4
COPPER PROTEIN	Q9ZUX4	AT2G27730	2
SELENIUM-BINDING PROTEIN 1	O23264	AT4G14030	3
LIPID TRANSFER PROTEIN 1	Q42589	AT2G38540	3
ACTIN 8/ACTIN 2	Q96293/Q96292	AT1G49240/AT3G18780	4 - 5
RIBOSOMAL PROTEIN L11	Q9LFH5/Q9FF52/ P50883	AT3G53430/AT5G60670/ AT2G37190	4 - 5
UDP-GLUCOSE PYROPHOSPHORYLASE 1	Q9M9P3	AT3G03250	3 - 7
ARABIDOPSIS THALIANA CYCLOPHILIN 1	Q42406	AT4G34870	4
EMBRYO SAC DEVELOPMENT ARREST 38/SELENIUM-BINDING PROTEIN 2	Q93WN0	AT4G14040	4
GLYCERALDEHYDE 3-PHOSPHATE DEHYDROGENASE A SUBUNIT 2	Q9LPW0	AT1G12900	4
LESION INITIATION 2	Q9LR75	AT1G03475	8
GLYCERALDEHYDE-3-PHOSPHATE DEHYDROGENASE C SUBUNIT	P25858	AT3G04120	2
PSA E1 KNOCKOUT	Q9S831	AT4G28750	2 - 4
GLUTATHIONE S-TRANSFERASE PHI 9	O80852	AT2G30860	5 - 9
RUBISCO SMALL SUBUNIT 3B/RUBISCO SMALL SUBUNIT 2B	P10798/P10797	AT5G38410/AT5G38420	4 - 5
HEAT SHOCK COGNATE PROTEIN 70-1	P22953	AT5G02500	2 - 5
CYCLOPHILIN 19, ROTAMASE CYP 3	Q38900	AT2G16600	3 - 5
PHOTOSYSTEM II SUBUNIT Q	Q41932	AT4G05180	5 - 6
GLUTAMATE-1-SEMIALDEHYDE 2,1-AMINOMUTASE 2	Q42522	AT3G48730	4 - 5
RAN NUCLEAR PROTEIN GTPASE 3/2/1	Q8H156/P41917/ P41916	AT5G55190/AT5G20020/ AT5G20010	5 - 7
2-CYS PEROXIREDOXIN A	Q96291	AT3G11630	5
METHIONINE ADENOSYLTRANSFERASE 4	Q9LUT2	AT3G17390	5
THYLAKOID FORMATION1	Q9SKT0	AT2G20890	5 - 8
METHIONINE SYNTHASE 2	Q9SRV5	AT3G03780	5 - 6
CATALASE 2	P25819	AT4G35090	6
METHIONINE SULFOXIDE REDUCTASE A4	P54150	AT4G25130	5 - 6
FIBRILLIN 4	Q9LW57	AT3G23400	6 - 7
CHLOROPLAST STEM-LOOP BINDING PROTEIN OF 41 KDA	Q9LYA9	AT3G63140	6 - 9
GLUTATHIONE S-TRANSFERASE TAU 19	Q9ZRW8	AT1G78380	6 - 10
GLYCERALDEHYDE 3-PHOSPHATE DEHYDROGENASE A SUBUNIT 1	P25856	AT3G26650	6 - 7
CYCLOPHILIN 20–3	P34791	AT3G62030	7 - 8
ASPARTATE AMINOTRANSFERASE 5	P46248	AT4G31990	7 - 14
O-ACETYLSERINE (THIOL) LYASE (OAS-TL) ISOFORM A1	P47998	AT4G14880	7 - 13
PHOSPHOGLYCERATE KINASE FAMILY PROTEIN 2	P50318	AT1G56190	7
GLUTATHIONE S-TRANSFERASE TAU 20	Q8L7C9	AT1G78370	7 - 10
PEROXIREDOXIN Q	Q9LU86	AT3G26060	5 - 7
NONPHOTOCHEMICAL QUENCHING 4, PHOTOSYSTEM II SUBUNIT S	Q9XF91	AT1G44575	5 - 7
PHOTOSYSTEM II REACTION CENTER PROTEIN A	P83755	ATCG00020	2 - 8
EMBRYO DEFECTIVE 1395,HOMOLOGY-DEPENDENT GENE SILENCING 1, MATERNAL EFFECT EMBRYO ARREST 58, S-ADENOSYL-L-HOMOCYSTEIN HYDROLASE 1,	O23255	AT4G13940	8 - 9
TRANSLATIONALLY CONTROLLED TUMOR PROTEIN	P31265	AT3G16640	5 - 8
BETA CARBONIC ANHYDRASE 2	P42737	AT5G14740	8
ASCORBATE PEROXIDASE 1	Q05431	AT1G07890	8 - 12
THIAMINE4	Q38814	AT5G54770	4 - 8
OXYGEN EVOLVING COMPLEX SUBUNIT 23 KDA	Q42029	AT1G06680	8
CHLOROPHYLL A/B BINDING PROTEIN 3/2/1	Q8VZ87/P0CJ48/ P04778	AT1G29910/AT1G29920/ AT1G29930	6 - 8
GLYCERATE KINASE	Q944I4	AT1G80380	8 - 10
GLYCOLATE OXIDASE 1	Q9LRR9	AT3G14420	8
PHOTOSYSTEM II SUBUNIT Q	Q9XFT3	AT4G21280	8 - 9
RIBOSOMAL L5P PROTEIN	O04603	AT4G01310	7 - 9
NUCLEOSIDE DIPHOSPHATE KINASE 1	P39207	AT4G09320	8 - 9
MONODEHYDROASCORBATE REDUCTASE 6	P92947	AT1G63940	9 - 11
NITRITE REDUCTASE 1	Q39161	AT2G15620	6 - 9
DEHYDROASCORBATE REDUCTASE 5	Q9FWR4	AT1G19570	9 - 14
ADP-RIBOSYLATION FACTOR 1/BFA-VISUALIZED EXOCYTIC TRAFFICKING DEFECTIVE 1/ATARFA1D	Q9LQC8/P36397/ P0DH91	AT1G23490/AT2G47170/ AT1G70490	8 - 9
CHLOROPLAST HEAT SHOCK PROTEIN 70–2	Q9LTX9	AT5G49910	6 - 9
RIBOSE 5-PHOSPHATE ISOMERASE,TYPE A PROTEIN	Q9S726	AT3G04790	6 - 9
PEROXIDASE 34, PEROXIDASE CB	Q9SMU8	AT3G49120	9 - 10
MITOCHONDRIAL MALATE DEHYDROGENASE 1/2	Q9ZP06/Q9LKA3	AT1G53240/AT3G15020	9 - 10
RIBULOSE BISPHOSPHATE CARBOXYLASE SMALL CHAIN 1A	P10795	AT1G67090	6 - 10
PROTOCHLOROPHYLLIDE OXIDOREDUCTASE B	P21218	AT4G27440	10 - 12
OXYGEN EVOLVING POLYPEPTIDE 1, MANGANESE-STABILIZING PROTEIN 1	P23321	AT5G66570	5 - 10
GLUTATHIONE S-TRANSFERASE PHI 2/GLUTATHIONE S-TRANSFERASE 16, GLUTATHIONE S-TRANSFERASE F3	P46422/Q9SLM6	AT4G02520/AT2G02930	9 - 10
ALDEHYDE DEHYDROGENASE 11A3	Q1WIQ6	AT2G24270	5 - 10
GLYCOLATE OXIDASE 2	Q9LRS0	AT3G14415	6 - 11
CYTOSOLIC NADP+-DEPENDENT ISOCITRATE DEHYDROGENASE	Q9SRZ6	AT1G65930	11 - 13
LIGHT HARVESTING COMPLEX OF PHOTOSYSTEM II 5	Q9XF89	AT4G10340	7 - 11
PEROXISOMAL NAD-MALATE DEHYDROGENASE 2	Q9ZP05	AT5G09660	11
ARGININE AMIDOHYDROLASE 2	Q9ZPF5	AT4G08870	10 - 11
GLYCINE CLEAVAGE T-PROTEIN FAMILY	O65396	AT1G11860	12 - 17
EPITHIOSPECIFIER MODIFIER 1	Q9LJG3	AT3G14210	12
PECTIN METHYLESTERASE 3	O49006	AT3G14310	8 - 13
NAD(P)-BINDING ROSSMANN-FOLD PROTEIN	O80934	AT2G37660	7 - 13
PHOSPHORIBULOKINASE	P25697	AT1G32060	12 - 13
HIGH CYCLIC ELECTRON FLOW 1	P25851	AT3G54050	11 - 13
CYTOSOLIC ISOFORM TRIOSE PHOSPHATE ISOMERASE	P48491	AT3G55440	10 - 13
ASCORBATE PEROXIDASE 4, THYLAKOID LUMEN 29,	P82281	AT4G09010	12 - 13
ATP SYNTHASE SUBUNIT 1	P92549	ATMG01190	11 - 13
HYDROXYMETHYLBILANE SYNTHASE	Q43316	AT5G08280	10 - 13
MONODEHYDROASCORBATE REDUCTASE 1	Q9LFA3	AT3G52880	13 - 18
FRUCTOSE-BISPHOSPHATE ALDOLASE 1	Q9SJU4	AT2G21330	8 - 13
KETOL-ACID REDUCTOISOMERASE	Q05758	AT3G58610	11 - 14
ALANINE:GLYOXYLATE AMINOTRANSFERASE	Q56YA5	AT2G13360	8 - 14
CHLOROPLAST RNA BINDING	Q9SA52	AT1G09340	13 - 14
PLASTID ISOFORM TRIOSE PHOSPHATE ISOMERASE	Q9SKP6	AT2G21170	11 - 14
LOW EXPRESSION OF OSMOTICALLY RESPONSIVE GENES 2	P25696	AT2G36530	15 - 17
CYSTEINE SYNTHASE 1	P47999	AT2G43750	13 - 15
FRUCTOSE-BISPHOSPHATE ALDOLASE 2	Q944G9	AT4G38970	15
GLYCINE DECARBOXYLASE P-PROTEIN 1	Q94B78	AT4G33010	8 - 15
PHOSPHOGLYCERATE KINASE 1	Q9LD57	AT3G12780	13 - 15
ALANINE-2-OXOGLUTARATE AMINOTRANSFERASE 1	Q9LR30	AT1G23310	15 - 20
SEDOHEPTULOSE-BISPHOSPHATASE	P46283	AT3G55800	16 - 17
CATALASE 3	Q42547	AT1G20620	13 - 16
HYDROXYPYRUVATE REDUCTASE	Q9C9W5	AT1G68010	15 - 16
GTP BINDING ELONGATION FACTOR TU FAMILY PROTEIN	Q8W4H7/Q8GTY0/ Q0WL56/P0DH99	AT1G07930/AT5G60390/ AT1G07920/AT1G07940	15 - 17
SERINE HYDROXYMETHYLTRANSFERASE 1	Q9SZJ5	AT4G37930	17 - 19
ATP SYNTHASE C1	Q01908	AT4G04640	10 - 18
COBALAMIN-INDEPENDENT METHIONINE SYNTHASE	O50008	AT5G17920	19 - 20
VARIEGATED 2/FTSH PROTEASE 8	O80860/Q8W585	AT2G30950/AT1G06430	10 - 19
RAB GTPASE HOMOLOG E1B	P17745	AT4G20360	17 - 19
GLUTAMINE SYNTHETASE 2	Q43127	AT5G35630	14 - 19
GLUCOSIDE GLUCOHYDROLASE 2	Q9C5C2	AT5G25980	12 - 19
SNOWY COTYLEDON 1	Q9SI75	AT1G62750	5 - 19
GLYCERALDEHYDE-3-PHOSPHATE DEHYDROGENASE B SUBUNIT	P25857	AT1G42970	20 - 21
ALLENE OXIDE SYNTHASE	Q96242	AT5G42650	15 - 20
CORONATINE INDUCED 1	Q9SUR6	AT4G23600	11 - 20
MITOCHONDRIAL ATP SYNTHASE BETA-SUBUNIT	P83484/P83483/ Q9C5A9	AT5G08690/AT5G08670/ AT5G08680	20 - 23
TRANSKETOLASE 1	Q8RWV0	AT3G60750	26 - 30
CHAPERONIN-60ALPHA	P21238	AT2G28000	18 - 28
ATP SYNTHASE SUBUNIT ALPHA	P56757	ATCG00120	21 - 30
FERREDOXIN-DEPENDENT GLUTAMATE SYNTHASE	Q9ZNZ7	AT5G04140	29 - 31
LIPOXYGENASE 2	P38418	AT3G45140	31 - 39
ATP SYNTHASE SUBUNIT BETA	P19366	ATCG00480	35 - 40

**Table 2.** Significant protein glycation changes in the 112 common targets during high light, heat, diurnal time-series, and rosette ageing

Light			
Name	ID	TAIR ID	Control/high light
RPI3	Q9S726	AT3G04790	–2.44
TPI	Q9SKP6	AT2G21170	–∞
**Heat**			
Name	ID	TAIR ID	Control/heat
TPI	Q9SKP6	AT2G21170	–∞
**Time-series**			
Name	ID	TAIR ID	08.00 h/24.00 h
AOC2	Q9LS02	AT3G25770	–1.7
ASP5	P46248	AT4G31990	2.9
BAS1	Q96291	AT3G11630	–1.62
CORI3	Q9SUR6	AT4G23600	–1.37
FTSH2	O80860	AT2G30950	1.68
OASB	P47999	AT2G43750	–1.59
PRK	P25697	AT1G32060	–0.937
PRXQ	Q9LU86	AT3G26060	–1.77
PURA	Q96529	AT3G57610	–1.24
RAN3	Q8H156	AT5G55190	2.27
**Rosette age**			
Name	ID	TAIR ID	4 weeks/6 weeks
A2	Q8W4H7	AT1G07930	1.92
CAT2	P25819	AT4G35090	–2.47
CICDH	Q9SRZ6	AT1G65930	–1.8
CTIMC	P48491	AT3G55440	–2.36
CYP18-4	Q42406	AT4G34870	–2.78
FBP	P25851	AT3G54050	–2.26
GGAT1	Q9LR30	AT1G23310	–2.99
GLU1	Q9ZNZ7	AT5G04140	–2.03
GSA2	Q42522	AT3G48730	1.77
LOX2	P38418	AT3G45140	–1.44
P25697	P25697	AT1G32060	–1.46
P83484	P83484	AT5G08690	1.2
PER34	Q9SMU8	AT3G49120	–1.93
RBCS-1A	P10795	AT1G67090	–3.74
RBCS-3B	P10798	AT5G38410	–2.38
TGG2	Q9C5C2	AT5G25980	–3.99
TL29	P82281	AT4G09010	–1.49

Values represent the label-free quantification log2 ratios for the different experiments at a significance of *P*<0.05. TPI was present in high light- and heat-stressed samples but absent in the controls

Gene Ontology (GO) analysis of the 112 target proteins revealed that the majority of these targets were classified as carrying out catalytic activity (82.5%) and thus may be considered as having an enzymatic function (Table 3). Furthermore, the majority of the proteins were located within the chloroplast (89.7%; Table 3). The few significant perturbations in the spectral counts and label-free quantification intensities in these 112 targets even under quite severe stress conditions (e.g. heat or light; Table 2) suggests that these proteins represent a base level of continually glycated proteins in plant cells and reveal that a high proportion of chloroplast enzymes are frequently glycated.

**Table 3.** Gene Ontology analysis of the core 112 proteins targeted for glycation in Arabidopsis

Term	Count	%	Fold enrichment	*P*-value
				Bonferroni
**Molecular function**				
Oxidoreductase activity	35	36.1	4.1	1.41E-10
Cofactor binding	19	19.6	7.2	2.94E-08
Catalytic activity	80	82.5	1.6	1.73E-07
Copper ion binding	12	12.4	9.3	1.76E-05
Peroxidase activity	9	9.3	12.7	1.59E-04
Ion binding	44	45.4	2	1.81E-04
Oxidoreductase activity	35	36.1	4.1	1.41E-10
Cofactor binding	19	19.6	7.2	2.94E-08
**Cellular compartment**				
Apoplast	64	66	33.6	1.18E-82
Chloroplast stroma	59	60.8	23.4	1.94E-65
Chloroplast	87	89.7	5.9	6.22E-57
Chloroplast envelope	49	50.5	23.4	2.37E-52
Thylakoid	30	30.9	37.8	5.29E-36
Chloroplast thylakoid	22	22.7	27.7	1.67E-22
Stromule	14	14.4	100.8	2.35E-21
**Biological process**				
Reductive pentose-phosphate cycle	12	12.4	114.4	1.67E-18
Oxidative photosynthetic carbon pathway	4	4.1	84.8	0.002832
Gluconeogenesis	5	5.2	68.1	1.67E-04
Photorespiration	8	8.2	30.5	1.72E-06
Glycolytic process	11	11.3	29.5	7.95E-10
Chlorophyll biosynthetic process	5	5.2	22.7	0.016572
Reductive pentose-phosphate cycle	12	12.4	114.4	1.67E-18

The top eight terms (fold enrichment) for biological process, cellular compartment, and biological process are shown. Analysis was carried out using The Database for Annotation, Visualization and Integrated Discovery (DAVID) v6.8 (Huang *et al.*, 2009*a*, *b*).

### Treatment-specific targets of glycation

We had initially hypothesized that stress treatments leading to oxidative stress and increased sugar levels would either significantly change specific protein targets or substantially increase levels of glycation within proteins. Yet, the 112 common target proteins remained stable during the stress treatments, with very few exceptions (see above; Table 3). However, comparisons also revealed unique glycation targets for all treatments (Fig. 4A), with few significant differences in the diurnal and drought experiments compared with their respective controls (Tables 4, 5). For the high light- and heat-specific glycation targets, however, no significant differences were observed.

**Table 4.** Specific proteins targeted for glycation unique to the diurnal experiment in Arabidopsis

Name	ID	TAIR ID	16.00 h versus 24.00 h	08.00 h versus 24.00 h	08.00 h versus 16.00 h
ADK1	Q9SF85-2	AT3G09820	***4.11***	2.16	–1.95
RPL23AB	Q9M3C3	AT3G55280	***2.63***	***2.21***	–0.418
ALDH5F1	Q9SAK4	AT1G79440	–0.492	***–1.46***	–0.973
MPPA2	O04308	AT3G16480	–1.3	***1.63***	***2.93***
Q0WW26	Q0WW26	AT4G34450	***–2.41***	–0.551	1.86
GLN1-3	Q9LVI8	AT3G17820	***–3.03***	–0.948	2.08
XYL1	Q9S7Y7	AT1G68560	–1.98	1.18	***3.16***
AGP31	Q9FZA2-2	AT1G28290	–1.49	***2.32***	***3.8***
P59259	P59259	AT5G59970 AT1G07660/ AT1G07820/ AT2G28740/ AT3G45930/ AT3G46320/ AT3G53730/ AT5G59690/	***–6.16***	***–4.96***	1.2

Label-free quantification log2 intensities, with those that are significant (*P*<0.05) shown in bold.

**Table 5.** Specific proteins targeted for glycation unique to the drought experiments in Arabidopsis

Name	ID	TAIR ID	Control (90% rSWC) versus drought (20% rSWC)
THM1	O48737	AT1G03680	–2.31
PDX11	O80448	AT2G38230	1.36
PsbB	P56777	ATCG00680	2.30
AIR12	Q94BT2	AT3G07390	1.69
CSY4	P20115-2	AT2G44350	–1.13
DHAR3	Q8LE52	AT5G16710	–2.00
EMB1144	P57720	AT1G48850	1.67
FDH1	Q9S7E4	AT5G14780	–2.18
FQR1	Q9LSQ5	AT5G54500	–1.78
GRF10	P48347	AT1G22300	–2.07
At14a	P0DI79	AT3G28300	–1.54
PetA	A4QKU4	ATCG00540	1.77
PPA6	Q9LXC9	AT5G09650	–2.19
Q9C7N5	Q9C7N5	AT1G29660	–1.57
TRXF1	Q9XFH8	AT3G02730	–1.68
RPL1	Q9LY66	AT3G63490	1.47
TRX5	Q39241	AT1G45145	–1.59
VHA-B1	P11574	AT1G76030	2.06

Values represent the label-free quantification log2 ratios (control/drought) at a *P*<0.05.

### Diurnal changes in glycation targets shows a spike in glycation levels at the end of the light period

Specific AGEs have previously been shown to change diurnally and in response to abiotic stress (Bechtold *et al.*, 2009; Paudel *et al.*, 2016), Overall, there were few significant changes in the112 common target proteins (see above; Table 2) or in time-specific target proteins (Table 4). Interestingly, while there were few significant differences in proteins targeted for glycation in a time-specific manner, the percentage of glycated peptides did increase, with an ~31% increase from 08.00 h to 16.00 h (Supplementary Fig. S3B). This suggests that the proteins targeted for glycation remain constant but the level of glycation within these proteins accumulates during the day, coinciding with an increase in sugar and starch levels (Supplementary Fig. S3A).

### Drought stress increases glycation of thioredoxin proteins associated with the regulation of Calvin–Benson cycle enzymes

In contrast to heat and high light stresses, there were 18 unique protein targets that significantly differed in abundance between well-watered (90% rSWC) and severely drought-stressed plants (20% rSWC; Table 5; Supplementary Fig. S5B; Bechtold *et al.*, 2018). Among these drought-specific targets were three thioredoxins, two present in the chloroplast (thioredoxin M1 and thioredoxin F1) and one present in the cytosol (thioredoxin H5; Table 5), and a glutathione *S*-transferase containing a thioredoxin-like fold (Table 5). In animals, thioredoxin activity is regulated by different post-translational modifications including glycation (Yuan *et al.*, 2010). This suggests that glycation of plant thioredoxins may potentially impact key functions of thioredoxin and thioredoxin-like proteins in regulating the structure and function of diverse target proteins.

### Glycation of recombinant GAPC1 and TPI enzymes leads to significant reduction in catalytic efficiency

**Fig. 5.**
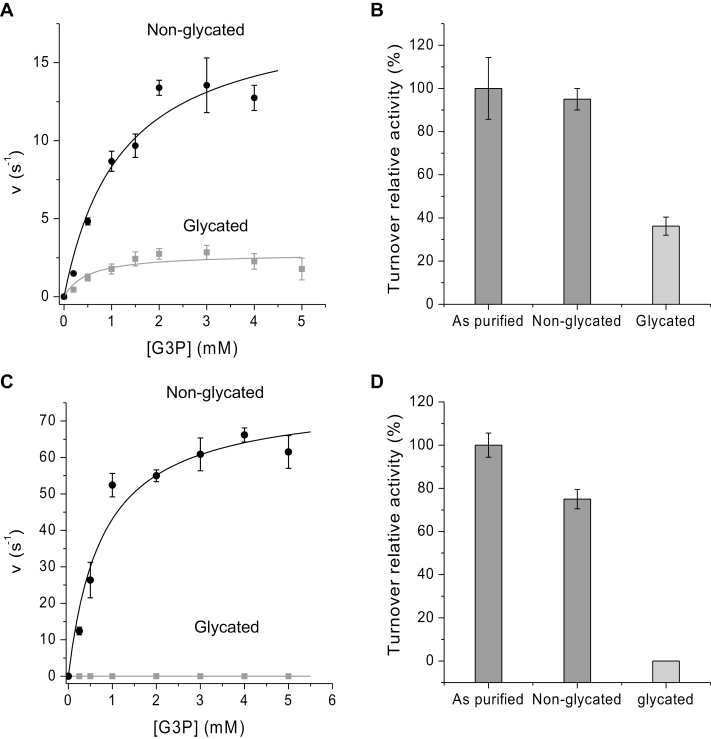
Steady-state enzyme kinetics of Arabidopsis glyceraldehyde-3-phosphate dehydrogenase (GAPC1) and triosephosphate isomerase (TPI). (A) Plots of turnover rate constants of TPI versus dl-glyceraldehyde-3-phosphate (G3P) substrate concentration. Non-glycated TPI shown in black and glycated TPI after 3 weeks in grey, with the data points fitted to the Michaelis–Menten equation to yield a *K*_m_ value and turnover rate (*k*_cat_). (B) The percentage relative turnover activity of purified TPI, non-glycated and glycated after 3 weeks. (C) Plots of turnover rate constants of GAPC1 versus G3P substrate concentration in 10 mM NaH_2_PO_4_/Na_2_HPO_4_, pH 8.5, 100 mM NaCl. Non-glycated GAPC1 shown in black and glycated GAPC1 after 3 weeks in grey, with the data points fitted to the Michaelis–Menten equation to yield a *K*_m_ value and turnover rate (*k*_cat_). (D) The percentage relative turnover activity of purified GAPC1, non-glycated and glycated after 3 weeks. *K*_m_, *k*_cat_, and *k*_cat_/*K*_m_ values are given in Table 6. All experiments were performed at 25 °C using a Hewlett-Packard 8453 diode-array spectrophotometer.

The above GO analysis suggested that ~82.5% of the 112 common target proteins were associated with catalytic function (Tables 1, 3). Among these, cytosolic and chloroplastic GAPC1 and GAPA2 (glyceraldehyde-3-phosphate dehydrogenase C1 and A2, respectively) and CTPI and TPI (cytosolic and chloroplastic triosephosphate isomerase, respectively) were identified. To investigate whether glycation affects enzyme activity and whether there is a difference between cellular compartments, cytosolic GAPC1 (accession no. P25858) and chloroplastic TPI (accession no. Q9SKP6) were chosen from the 112 protein targets for further testing *in vitro*. Purified recombinant enzymes migrated as single bands on a denaturing polyacrylamide gel consistent with their molecular mass (~39 kDa for GAPC1 and ~29 kDa for TPI) (Supplementary Fig. S6A). GAPC1 and TPI were glycated with glucose at 37 °C (see the Material and methods). Following a 3 week incubation with and without glucose, glycated proteins were enriched using BAC. Importantly, control samples incubated without glucose also displayed a small glycated peak, suggesting that a basal level glycation also occurred in *E. coli*, and that glycated residues were stable during a 3 week incubation period at 37 °C (Supplementary Fig. S6B). Incubation with glucose led to a substantial increase in the glycation peak (Supplementary Fig. S6B). Activity assays for GAPC1 and TPI (see the Materials and methods) were conducted under steady-state conditions using (i) purified enzyme; (ii) buffer only-treated enzyme; and (iii) glycated enzyme. From steady-state kinetics, TPI displayed Michaelis–Menten kinetics for G3P with *k*_cat_, *K*_m_, and *k*_cat_/*K*_m_ (Table 6) in a similar range to previously observed *K*_m_ values for TPI and other plant triosephosphate isomerases (Turner *et al.*, 1965; Tang *et al.*, 1999; Chen and Thelen, 2010; Enriquez-Flores *et al*., 2011; Zaffagnini *et al.*, 2014; Dumont *et al.*, 2016; Lopez-Castillo *et al*., 2016). Following incubation at 37 °C with buffer only, TPI displayed similar kinetics with 5% lower turnover than the relative turnover (*k*_cat_/*K*_m_ value) of the ‘as-purified’ enzyme sample (Fig. 5A). However, once incubated with glucose at 37 °C for 3 weeks, the catalytic efficiency of TPI decreased by 63.1% (Fig. 5A, B; Table 6). The activity of GAPC1 displayed Michaelis–Menten kinetics (Table 6) but, unlike for TPI, *k*_cat_, *K*_m_, and *k*_cat_/*K*_m_ have not been previously reported. When GAPC1 was incubated at 37 °C with buffer only, the activity decreased by 25%. However, incubation of GAPC1with glucose for 3 weeks resulted in a completely inactive enzyme (Fig. 5C, D). Therefore, the catalytic efficiencies of both GAPC1 and TPI were inhibited when non-enzymatically glycated, with GAPC1 showing a much more dramatic reduction.

**Table 6.** Steady-state kinetic parameters for Arabidopsis enzymes glyceraldehyde-3-phosphate dehydrogenase (GAPC1 at pH 8.5) and triosephosphate isomerase (TPI at pH 7.0)

Enzyme	pH	*K*_m_ (mM)	*k*_cat_ (s^–1^)	*k*_cat_/*K*_m_ (M^–1^ s^–1^)
TPI non-glycated	7.0	1.21 (±0.39)	18.37 (±2.25)	1.5 × 10^4^
TPI glycated	7.0	0.5 (±0.30)	2.77 (±0.39)	5.54 × 10^3^
GAPC1 non-glycated	8.5	0.77 (±0.21)	76.4 (±5.91)	9.9 × 10^4^
GAPC1 glycated	8.5	0	0	0

*K*_m_, *k*_cat_, and *k*_cat_/*K*_m_ were determined from Michaelis–Menten plots.

### MS reveals different glycation patterns of GAPC1 and TPI

**Fig. 6.**
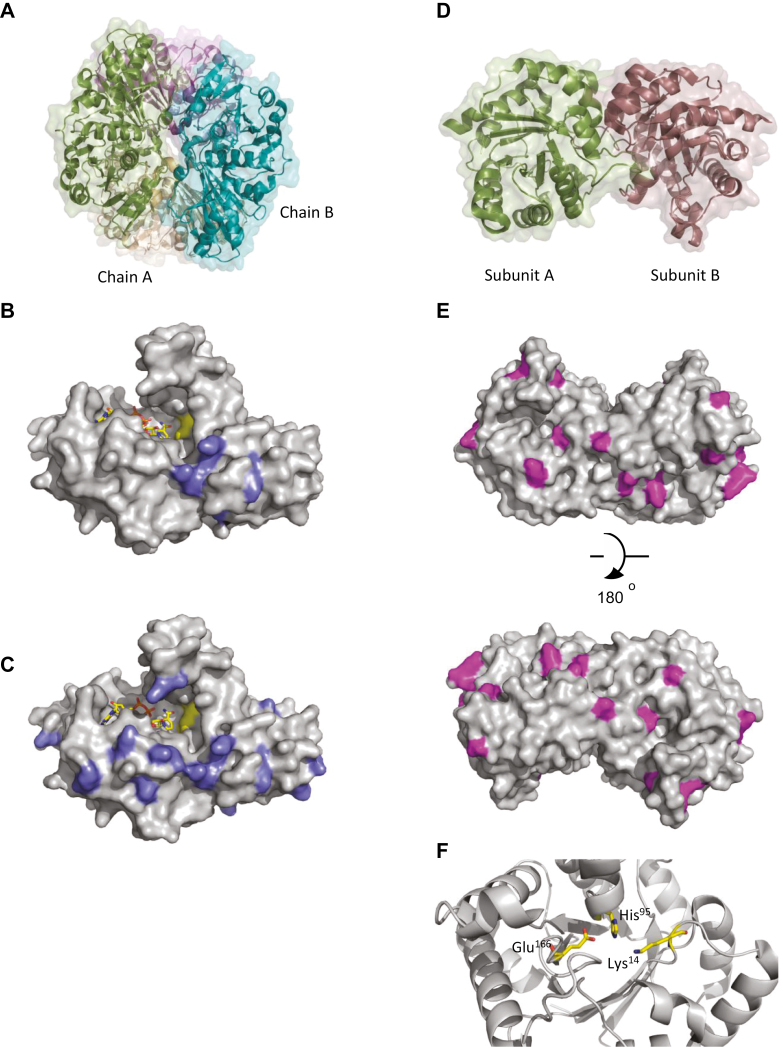
Structure of Arabidopsis enzymes glyceraldehyde-3-phosphate dehydrogenase C1 (GAPC1) and triosephosphate isomerase (TPI). (A) Biological tetramer assembly of GAPC1; each molecule is coloured differently (Chain A, white; Chain B, teal; symmetry-related molecules, green and purple). (B) Monomer of GAPC1 as purified from *E. coli* with the surface represented as grey, NAD+ shown in yellow sticks, and glycation sites illustrated on the surface in purple. (C) GAPC1 after 3 weeks glycation with 400 mM glucose; NAD+ is shown as yellow sticks and glycation sites as purple on a grey surface representation [PDB 4Z0H (Zaffagnini *et al.*, 2016)]. (D) Biological homodimeric assembly of TPI: subunit A in green and subunit B in dark red. (E) Surface representation of TPI with glycation sites indicated in pink in both purified and glycated TPI. (F) Catalytic active site of TPI with the main chain shown in grey cartoon and the active site residues (Lys14, Glu166, and His96) shown as yellow sticks [PDB entry 4OHQ (Lopez-Castillo *et al*., 2016)].

**Fig. 7.**
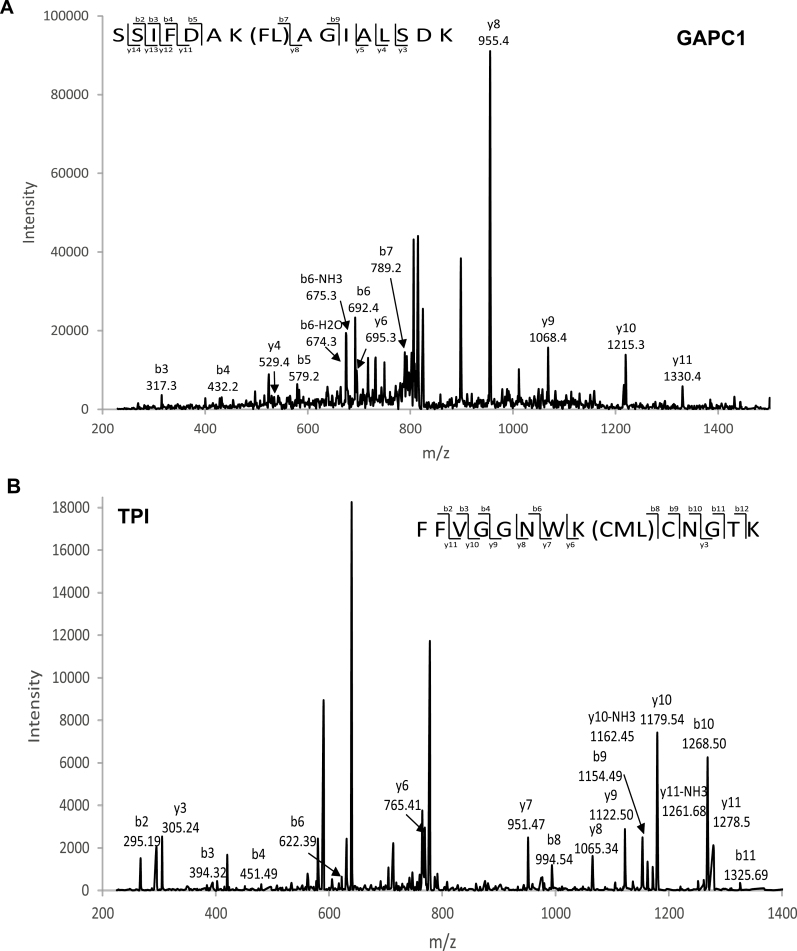
MS/MS data confirming protein glycation sites. (A) Tandem mass spectra of *m/z* 699.57 corresponding to the y122+ ion of the tryptic AGE-modified peptide SSIFDAK(FL)AGIALSDK, representing glyceraldehyde-3-phosphate dehydrogenase C1 (GAPC1), and (B) tandem mass spectra of the tryptic AGE-modified peptide FFVGGNWK(CML)CNGTK, representing triosephosphate isomerase (TPI).

To assess the changes in glycation sites and to gain an insight into why the catalytic activity of GAPC1 was completely abolished while TPI was able to maintain nearly 40% of its activity, we analysed specific glycation sites using MS. The acquired MS/MS spectrum positively identified GAPC1 and TPI (GAPC1 measured mass of 39077 Da, and TPI measured mass of 29273 Da). The GAPC1 protein contains 338 amino acids with 3.8% Arg and 9.8% Lys residues. We identified 78 peptides corresponding to GAPC1 of which 40 contained at least one of the modifications we searched for (CEL, CML, FL, G-H1, and MG-H1). The number of glycation sites increased from 6 in the non-glycated protein sample to 20 in the glycated sample. Furthermore, the number of glycated peptides increased from 10 to 69 (3% to 19% of the total amino acid count) from the non-glycated to the glycated GAPC1 protein sample. The glycation sites were mapped onto a single monomer of GAPC1 from the biologically relevant tetramer in solution (Fig. 6A) [PDB 4Z0H (Zaffagnini *et al.*, 2016)] by determining the probability with which the modification was present (Fig. 6). The modification patterns identified few glycation sites near the active site before incubation with glucose (Fig. 6B). In contrast, after glucose treatment, a significantly higher prevalence of seemingly random glycation sites was observed surrounding the active site of GAPC1 (Fig. 6C). This increase in glycation sites around the active site could explain the complete reduction in catalytic efficiency. An example of an FL-modified glycated peptide SSIFDAK(FL)AGIALSDK, from GAPC1 is shown in Fig. 7A. This peptide was not present in the non-glycated protein but increased to 11 peptides identified after glucose incubation.

The chloroplast-localized TPI functions as a homodimer and displayed a different glycation pattern from GAPC1 (Fig. 6D) [PDB entry 4OHQ (Lopez-Castillo *et al*., 2016)]. TPI contains 254 amino acids with 3.8% Arg and 7.3% Lys. We identified 57 peptides of which 28 contained a glycation site. Thirteen glycation sites were identified for both non-glycated and glycated samples, with the non-glycated protein containing one extra site at residue Lys139 (Fig. 6E). Interestingly, TPI displayed a much higher proportion of glycation directly purified from *E. coli* (8% compared with 3% in GAPC1) and, once glycated, the percentage increased from 8% to 13% (Fig. 6E). In total, 12/13 Lys and 1 Arg glycation site were identified within the protein, with these sites remaining the same before and after glycation (with the exception of Lys139). Instead, the abundance of glycated peptides at these sites increased following glycation (Fig. 6E). This suggests that the chloroplast enzyme experiences a higher level of glycation under the same conditions as the cytosolic enzyme, when expressed in *E. coli*. Lys14 within the active site of TPI was glycated, containing a CML when directly purified from *E. coli* (Fig. 6F). An example MS/MS spectrum of the peptide containing the CML modification (FFVGGNWK(CML)CNGTK) on Lys14 of TPI can be seen in Fig. 7B. Importantly, the number of this glycated peptide remained constant after glycation, which suggests that the lowered activity for TPI was not due to increased glycation of the active site Lys residue.

## Discussion

### General glycation levels during abiotic stress and rosette ageing

Previous research revealed that up to 502 proteins are targets for glycation in Arabidopsis leaves under various conditions (Bilova *et al.*, 2016). We reasoned that perturbations of primary metabolic pathways through stress treatments that lead to increases in sugar levels and ROS (i.e. H_2_O_2_) formation may also lead to altered glycation profiles in plants, as has been previously shown for specific AGE modifications (Bechtold *et al.*, 2009). Furthermore, in yeast and mammalian cells, high levels of glucose are associated with toxicity and pathogenesis through increased ROS production via glucose auto-oxidation and metabolism (Bonnefont-Rousselot, 2002; Russell *et al.*, 2002), and consequently experiments were chosen that would alter accumulation of sugar levels and oxidative stress production. We detected 460, 165, 181 and 527 glycated proteins for diurnal, heat, light, and drought responses, respectively, of which 112 proteins were common between the different treatments (Table 1). Interestingly, the short-term stress treatments, such as heat and high light, despite leading to significant oxidative stress (Fig. 2A–C) and increases in soluble sugars (Fig. 3A), did not result in a broad significant increase in the level of glycation within the 112 core targets, or in the emergence of many stress-specific glycation targets (Tables 1, 4). Most of the significant changes were observed during rosette ageing, drought, and the diurnal time-series, which were associated with less severe but probably more sustained changes in sugar, ROS, and glyoxalase I activity levels (Figs 2B, D–F; Supplementary Fig. S5A). This aligns with the role of protein glycation in the loss of seed viability during long-term seed storage (Wettlaufer and Leopold, 1991; Murthy and Sun, 2000; Murthy *et al.*, 2003), which is associated with sugar hydrolysis, accumulation of glucose, and lipid peroxidation in both accelerated and naturally aged seeds (Murthy and Sun, 2000; Murthy *et al.*, 2003). Seed ageing has also been closely connected to the formation of free radicals, oxidative stress, and a reduction in antioxidant capacities (Kranner *et al.*, 2006, 2010; Roach *et al.*, 2010), and suggests that the release of sugars and the accumulation of early and late stage glycation products may propagate free radical production. More recently, the age-related changes in the Arabidopsis glycation profile of proteins have been investigated, and only minimal changes in protein abundance were found. It was therefore suggested that glycation targets are conserved, with only a limited number of proteins being glycated during ageing (Bilova *et al.*, 2017). Minimal changes in protein abundance corroborate the findings from our diverse experiments, including a range of abiotic stress treatments (Paudel *et al.*, 2016; Bilova *et al.*, 2017). Yet direct comparison of protein targets, where available, indicated that there was overall little overlap between osmotic stress- and age-specific targets (Paudel *et al.*, 2016; Bilova *et al.*, 2017) with our 112 common, drought- and development-specific targets (Supplementary Fig. S7).

The identification of a number of significant AGE sites during plant ageing led to the proposal of ‘glycation hotspotsֹ within the plant proteome (Bilova *et al.*, 2017), which were in parts of the proteins essential for enzyme catalysis, activity, and interaction with other molecules (Bilova *et al.*, 2017). Glycation hotspots have also been shown for some mammalian proteins, such as serum albumin (Barnaby *et al.*, 2011) and recombinant antibodies (Zhang *et al.*, 2008), but whether hotspots serve a specific function is unclear. Intriguingly, the number of glycated proteins identified under low stringency conditions was higher when compared with applying a more stringent filter (Supplementary Table S3). However, the number of identified peptides and modification sites was reduced significantly when applying a more stringent cut-off (see the Results). The dramatic decrease in protein numbers and modification sites with increased filter stringency highlights the somewhat random nature of glycation within plant cells. In addition, due to the great variety of glycation products (Baynes *et al.*, 1989), it was not possible to search all of the glycation products present in our samples fully. However, we are confident that all identified proteins were specifically glycated due to their fractionation on the BAC and exclusive hybridization with glycation-specific antibodies (Fig. 1). More importantly, the 112 identified common targets were subject to more stringent filtering criteria, indicating a much greater consistency of glycation amongst this core set of proteins across the many different treatments.

Within the 112 common targets, we did observe increases in the number of glycated peptides during diurnal changes, with the highest number of glycated peptides occurring at the end of the light period (Supplementary Fig. S3), coinciding with the highest carbohydrate concentrations (Supplementary Fig. S3). To establish whether this could be related to particular glycation hotspots in proteins, we subsequently analysed two well-known recombinant plant enzymes for glycation sites. The results indicated that TPI may contain glycation hotspots, maintaining identical glycation between control and severely glycating conditions (Fig. 6D–F), while in GAPC1 the glycation pattern appeared to be much more random, with many more sites emerging under severely glycating conditions (Fig. 6A–C, discussed below).

### Impact of glycation on enzyme activity in different subcellular compartments

In animals, glycation of GAPDH has been shown to reduce catalytic efficiency dramatically (Muronetz *et al.*, 2016). We therefore selected chloroplastic TPI and cytosolic GAPC1 enzymes as part of the 112 core glycation targets (Table 1), and demonstrated that glycation affected enzyme activities, although with different sensitivities (Fig. 5). This could suggest that the subcellular localization of both enzymes impacts on the resilience to glycation, where the enzyme present in the cytosol is more susceptible to glycation than the chloroplast-located enzyme (Fig. 5). Alternatively, the existence of glycation hotspots versus random glycation may be due purely to the different three-dimensional protein structures and neighbouring amino acids (Sáenz-Suárez *et al.*, 2016; Bilova *et al.*, 2017) leading to site-specific increases of glycation levels, independent of subcellular localization and functionality. While it has previously been shown that glycation leads to a 70% decrease in Rubisco activity and increased protease susceptibility when incubated with the potent glycation agent ascorbate (Yamauchi *et al.*, 2002), it is not clear whether specific glycation hotspots emerge under those conditions. Recently, reduced chloroplast TPI activity was shown to lead to stunted and chlorotic seedlings that accumulated dihydroxyacetone phosphate (DHAP) and MG (Chen and Thelen, 2010), a highly reactive glycating agent. An increased resilience of this enzyme towards glycation would therefore be paramount to maintain cellular function.

Intriguingly, the substrates responsible for glycation, notably MG, hexoses, ascorbate (Yamauchi *et al.*, 2002), and sugar-phosphates (Fortpied *et al.*, 2005), are all produced and/or present at high concentrations within the chloroplast. In addition, the only known protective enzyme, fructosamine 3-kinase (FN3K), capable of reversing early glycation products (Szwergold *et al.*, 2001) is also located within the chloroplast exhibiting a 700 times higher activity than the equivalent enzyme in human erythrocytes (Fortpied *et al.*, 2005). Together with the majority of targets being chloroplast located (89.7%; Table 3), this suggests that chloroplast enzymes may not only be extensive targets for glycation, but may also have evolved to withstand increased glycation pressures. From the limited data available on glycation of plant proteins *in vivo*, it appears that chloroplast enzymes are able to maintain some degree of activity (Fig. 5A, C; Yamauchi *et al.*, 2002), while the cytosolic enzyme GAPC1 does not (Fig. 5B, D). Interestingly, TPI is absent in control samples and only emerges as a glycation target under high light and heat stress treatments, indicating minimal glycation under control conditions (Table 2). In comparison, GAPC1 is present both in the controls and in high light or heat treatments, and even though the degree of protein glycation cannot be assessed *in vivo* we have shown *in vitro* that glycation severely affects catalytic turnover. Further analysis of chloroplast and cytosolic enzymes is required to test this hypothesis.

### Drought-specific targets are linked to redox regulation of the Calvin cycle and abiotic stress responses

Among the drought-specific glycation targets, few are directly linked to abiotic stress responses and/or signalling. However, we identified some interesting functions and regulatory capacities that may impact on wider signalling pathways. Among the proteins with increased glycation levels under drought conditions were several enzymes with known regulatory functions (Table 5) For example, the plastidial pyrophosphatase is a glycation target (AtPPa6, At5g09650, Table 5), and it has previously been shown that altering a PPa6 homologue affects primary metabolism in tobacco. Furthermore, PPa6 gene silencing resulted in drought sensitivity due to reduced abscisic acid content (George *et al.*, 2010). Consequently, the removal of PPi has been suggested to play an important role in maintaining plastidial metabolism (George *et al.*, 2010). Increased glycation of PPa6 under drought stress and the potential reduction of enzyme activity could therefore contribute to physiological drought responses. Similarly, At14a, a small transmembrane protein, showed increased glycation levels during drought stress (Table 5, At3g28300), and the close homologue At14a-Like1 protein was linked to plant growth and proline accumulation under drought conditions involving protein disulphide isomerase 5 and NAI2 (Kumar *et al.*, 2015). The increase of glycation in three thioredoxins under drought conditions could indirectly have a much wider impact through their specific target proteins, especially since thioredoxin activity is regulated by different post-translational modifications and reduction of thioredoxin activity contributes to organ injury in many diseases (Sano *et al.*, 2002; Tsutsui *et al.*, 2003; Kondo *et al.*, 2004). Recently, the reduction of thioredoxin 1 activity through glycation was demonstrated to play a critical role in lipo-polysaccharide-related cell death (Yuan *et al.*, 2010), and thioredoxin-1 glycation was shown to be irreversible, resulting in permanent inactivation of this important signalling protein (Yuan *et al.*, 2010).

In plants, thioredoxins participate in the regulation of protein targets involved in plant development and in response to (a)biotic stresses, carry out redox sensing and signal transduction functions (Rouhier and Jacquot, 2005), and participate in the repair of oxidized proteins during environmental stress (Vieira Dos Santos and Rey, 2006).

For example, thioredoxin f1 is essential for the light-dependent reduction of specific Calvin–Benson enzymes such as fructose bisphosphatase (FBPase), sedoheptulose-1,7-biphosphatase (SBPase), phosphoribulokinase (PRK), glyceraldehyde-3-phosphate dehydrogenase (GAPDH), and Rubisco activase (Michelet *et al.*, 2013; Nikkanen *et al.*, 2017). Importantly, many of the thioredoxin f downstream targets are among the 112 conserved continually glycated proteins, but did not significantly increase during drought stress (Table 1), suggesting that specific glycation of a small number of key regulatory enzymes under stress may affect a greater number of downstream proteins and physiological processes, such as the down-regulation of photosynthesis during drought stress (Bechtold *et al.*, 2016).

### Conclusions

In this study, we demonstrated that glycation proceeds spontaneously in plants already under relatively benign growth conditions, resulting in many common glycation targets under different growth conditions. Glycation targets are largely located within the chloroplast, the site of production of high levels of sugars during photosynthesis, daily sugar fluctuations, and increased sugar levels during stressful conditions, particularly drought. This is analogous to animal systems where glycation is also associated with energy-producing metabolic processes, and non-enzymatic glycation together with free radical oxidation are considered to be the cause of many diabetes-related complications (see the Introduction). Intriguingly, glycated peptides have been associated with signalling processes leading to transcriptional changes in animal systems (see the Introduction). Whether glycated proteins and/or their breakdown products play a role in signalling responses in plants remains to be investigated. However, with the knowledge of specific targets, we can begin to unravel physiological functions with regard to specific plant responses.

## Supplementary data

Supplementary data are available at *JXB* online.

**Fig. S1.** Enrichment for glycated peptides using boronate affinity chromatography before and after alkaline hydrolysis.

**Fig. S2.** The effect of high light and heat on the Chl *a* efficiency.

**Fig. S3.** Carbohydrate analysis of diurnal period experiments in Arabidopsis.

**Fig. S4.** Mass spectrum of a glycated peptide from PSBP1 oxygen-evolving enhancer protein 2-1.

**Fig. S5.** Volcano plots from DEP R analysis of glycated proteins from Arabidopsis.

**Fig. S6.** SDS–PAGE gel analysis of GAPC1 and TPI, and boronate affinity chromatography of TPI from Arabidopsis.

**Fig. S7.** Comparison of glycated protein targets with published data sets (Bilova *et al*., 2016, 2017; Paudel *et al.*, 2016).

**Table S1.** The raw mass spectrum data for the protein groups identified from the time-series experiment in Arabidopsis.

**Table S2.** The raw mass spectrum data for the protein groups identified from the high light, heat, and drought experiments in Arabidopsis.

**Table S3.** Summary of glycated proteins, peptides, and modification sites for CML/CMA, M-GH1, GH1, and FL

## Supplementary Material

Supplementary File 1

Supplementary File 2
