# Supplementary material for: Profiling of advanced glycation end products uncovers abiotic stress-specific target proteins in Arabidopsis
Source: J Exp Bot. 2018 Nov 5;70(2):653–70. doi: 10.1093/jxb/ery389 (PMC6322573; doi:10.1093/jxb/ery389)
Supplement: Supplementary File 2 [file ery389_suppl_supplementary_file_2.pdf]

A

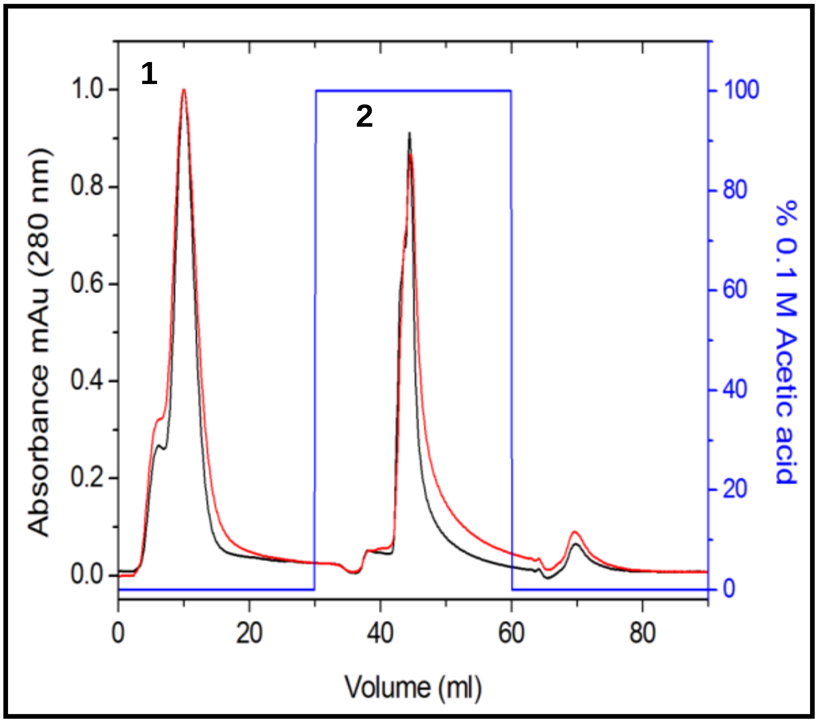

B

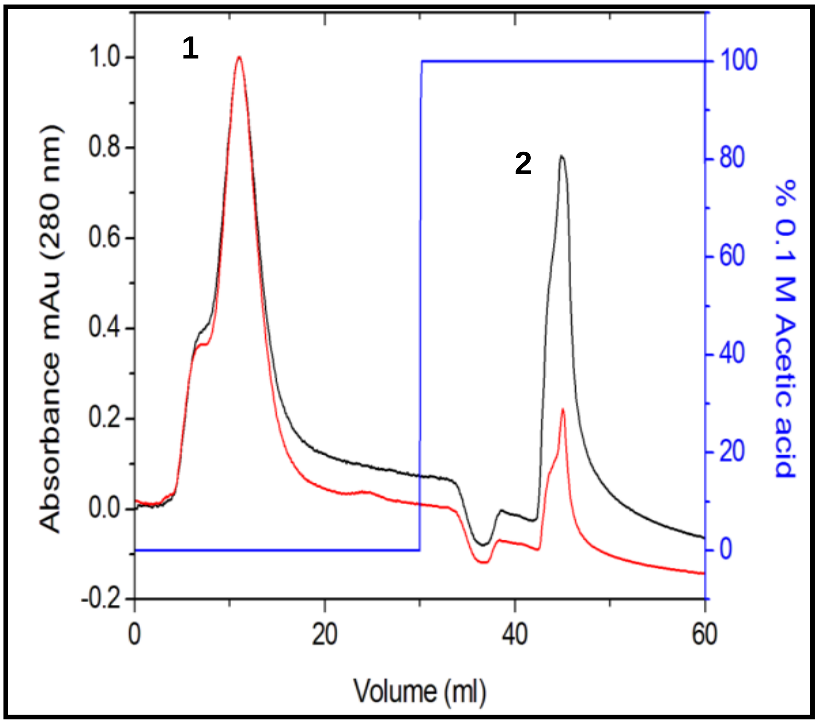

Supplemental Figure 1 Chaplin et al 2018

Figure S1. Enrichment for glycated peptides using boronate affinity chromatography before and after alkaline hydrolysis. **A –** glycated BSA with 400 mM glucose profile prior (black) and post (red) alkaline hydrolysis with 0.1 M NaOH at 45 °C for 6 hours and **B –** total Arabidopsis leaf extracts prior (black) and post (red) alkaline hydrolysis with 0.1 M NaOH at 45 °C for 6 hours. Peak 1 – glycated peptides not bound to column, peak 2 – glycated peptides bound to column and eluted in 0.1 M acetic acid. The blue line indicates the gradient of 0.1 M acetic acid applied to the column.

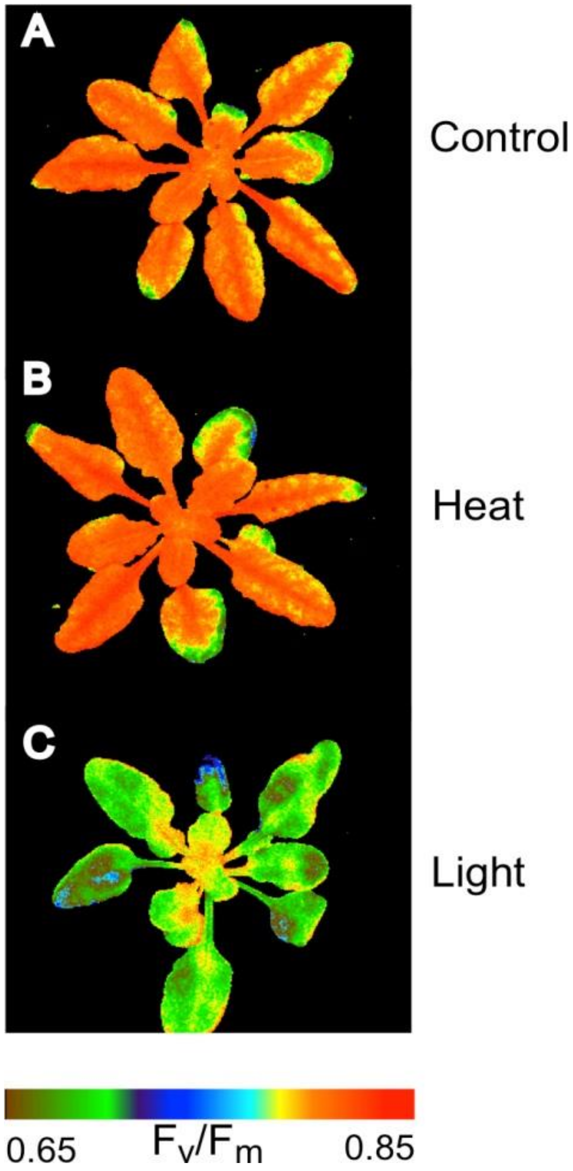

Supplemental Figure 2 Chaplin et al 2018

Figure S2. The effect of high light (IsoLight 850 µmol m^{-2} s^{-1}) and heat (37 ^{o}C, 78 % humidity) on the chlorophyll *a* fluorescence parameter F_v/F_m in WT Col-0 *A. thaliana* plants using a fluorescence-imaging instrument (Fluorimager, Technologica, Colchester UK). The fluorescent images shown are representative responses of all 6 biological replicates.

A

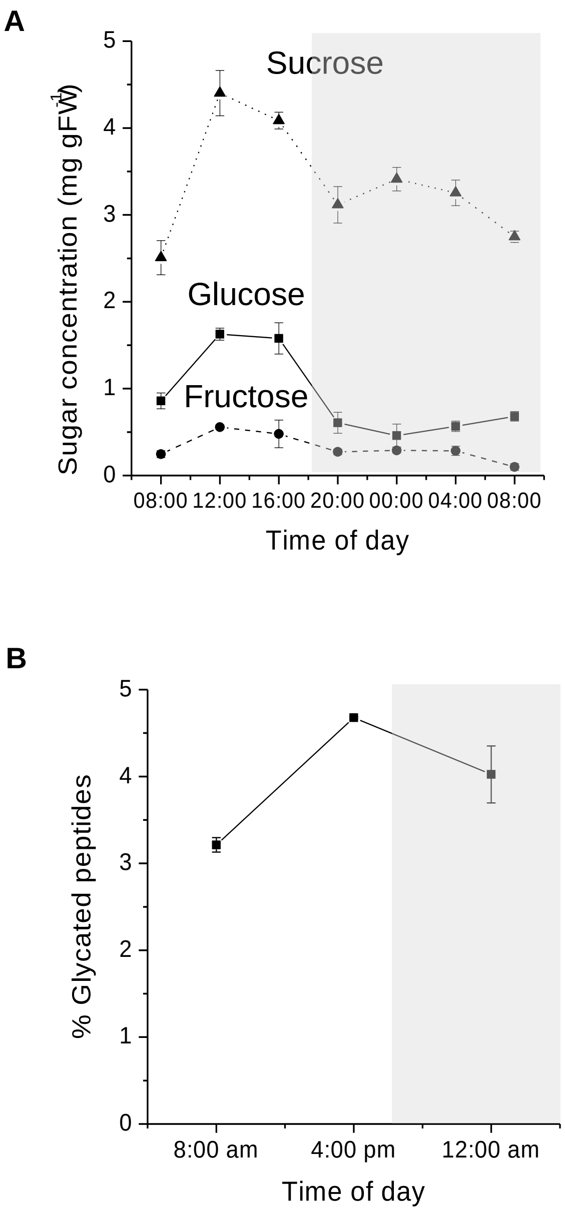

Supplemental Figure 3 Chaplin et al 2018

Figure S3. Carbohydrate analysis of time series experiments in *A. thaliana*. A) Soluble sugar concentrations of glucose (squares, solid line), fructose (circles, dashed line) and sucrose (triangles and dotted line) over a period of 24 h measured according to Stitt and Quick, 1989 (ref). B) The percentage of glycated peptides taken from the proteomic analysis of the time series experiment.

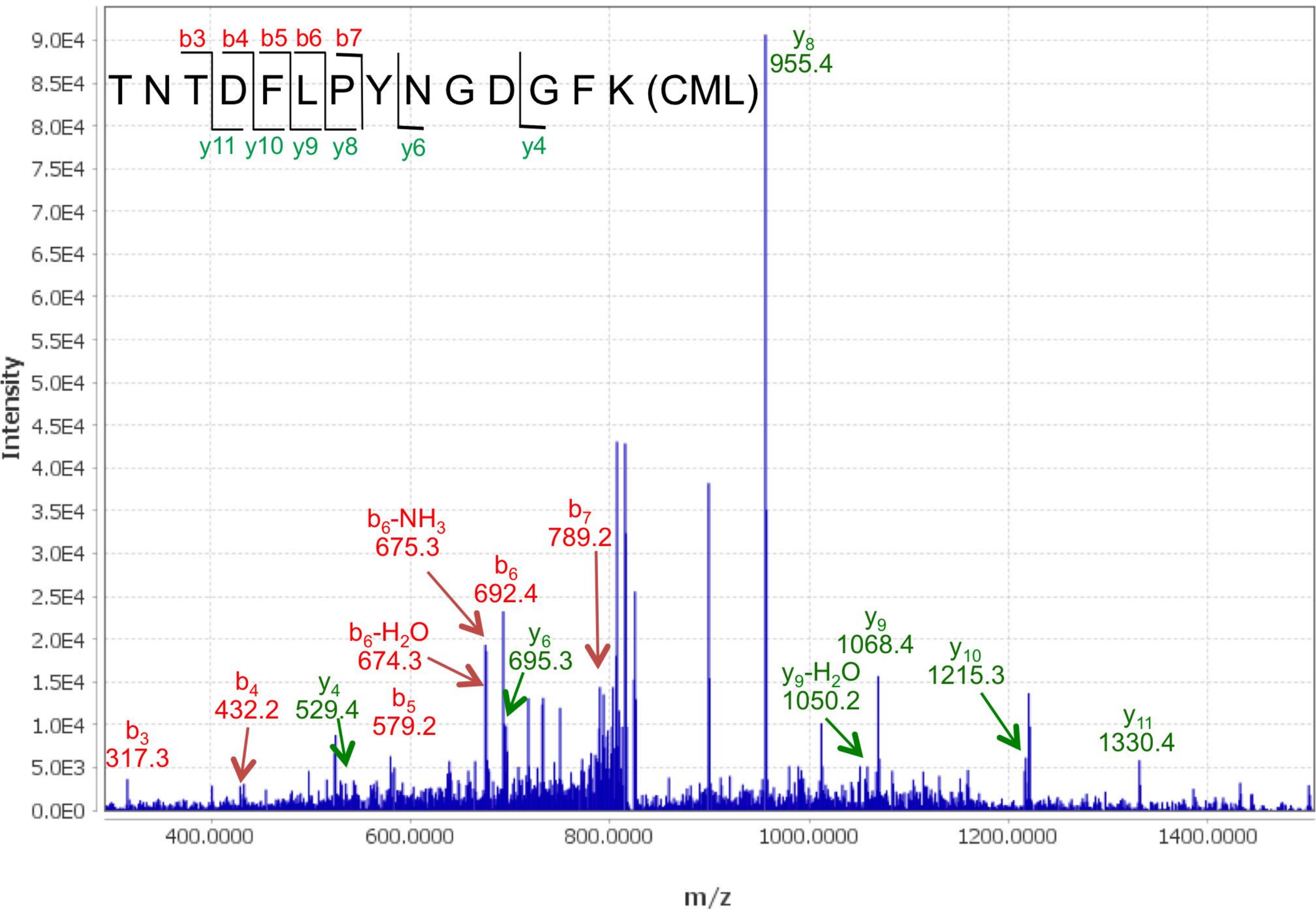

Supplemental Figure 4 Chaplin et al 2018

Figure S4. MS/MS data confirming protein glycation in *A. thaliana* proteins. Tandem mass spectra of m/z 955.4 corresponding to the y8$^+$ ion of the tryptic AGE-modified peptide TNTDFLPYNGDGFK(CML), representing the PSBP1 Oxygen-evolving enhancer protein 2-1, chloroplastic.

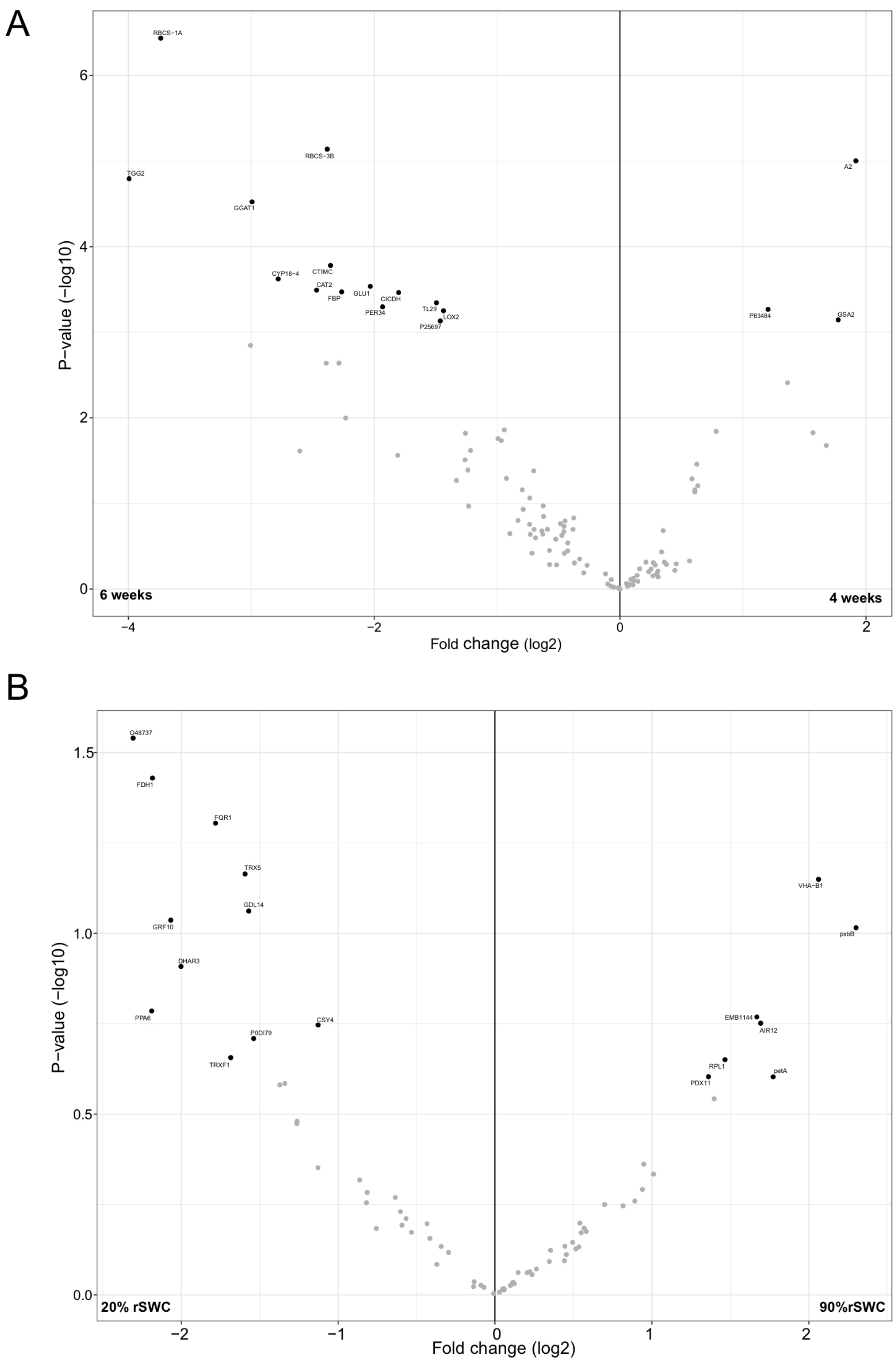

Supplemental Figure 5 Chaplin et al 2018

Figure S5. Volcano plots from DEP R analysis of glycated proteins from *A. thaliana*. **A** - Volcano plot showing significantly altered proteins during rosette development at 4- and 6 weeks in the 112 common protein targets. **B** - Volcano plot showing significantly altered proteins of drought stress specific protein targets. 20% rSWC – severe drought stressed, 90% rSWC well-watered plant material.

A

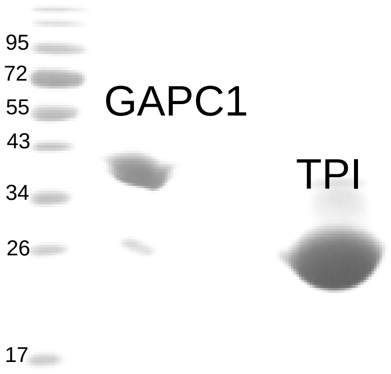

B

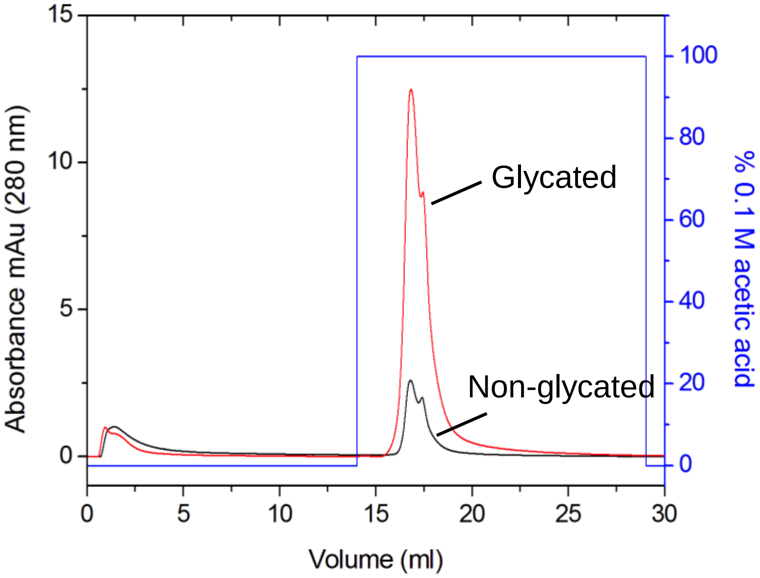

Supplemental Figure 6 Chaplin et al 2018

Figure S6: *A. thaliana* enzymes glyceraldehyde-3-phosphate dehydrogenase (GAPC1) and Triosephosphate Isomerase (TPI) recombinant expression in *E. coli*. **A -** SDS-PAGE gel analysis of purified GAPC1 and TPI consistent with their molecular mass (~39 kDa GAPC1 and ~29 kDa TPI). **B -** Boronate affinity chromatography chromatogram of TPI showing absorbance peaks (mAu) at 280 nm of as purified (Non-glycated, black) and 3 weeks after glycation (glycated, red) with the glycated peak eluted after 0.1 M acetic acid increasing after glycation.

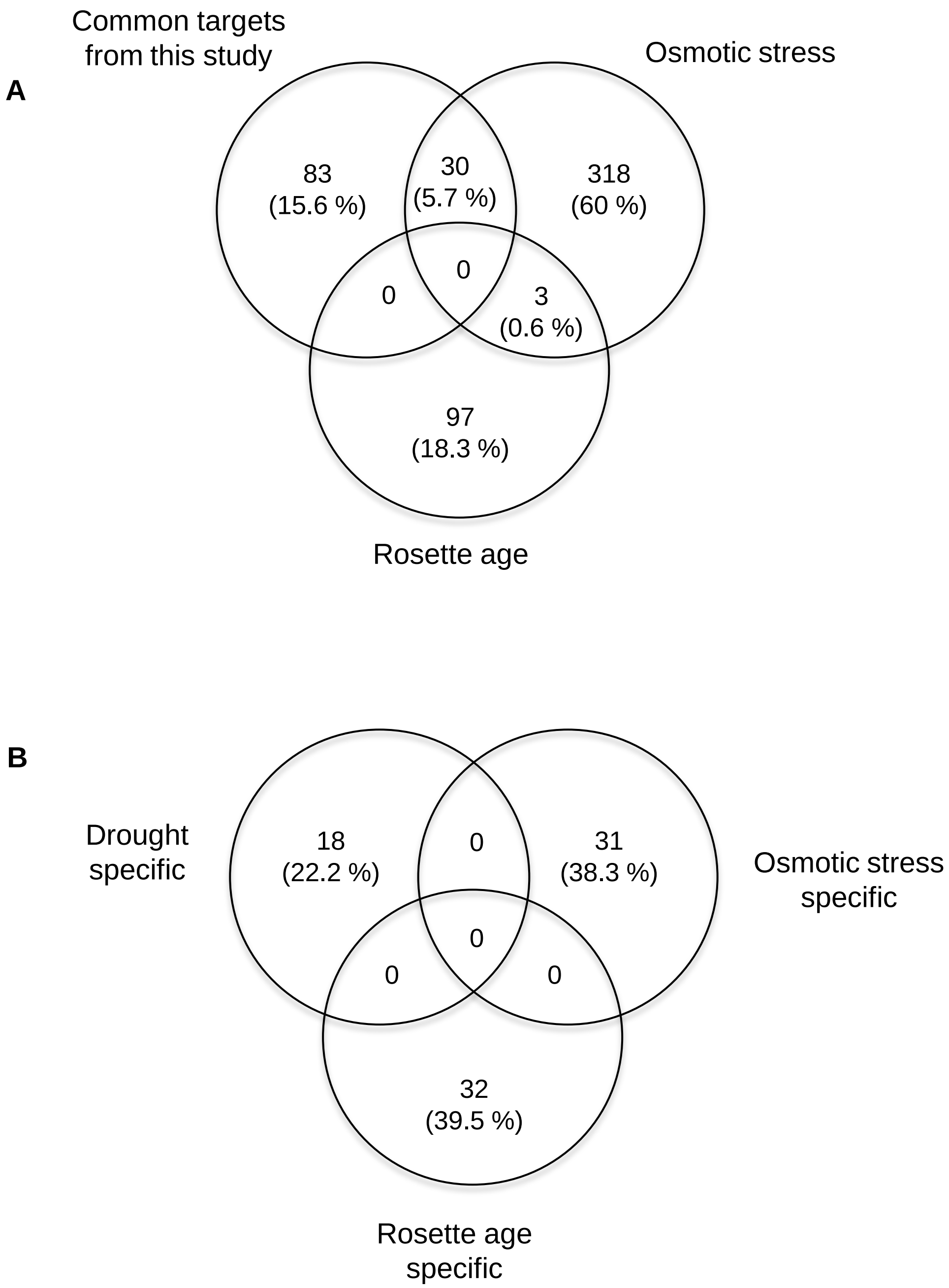

Supplemental Figure 7 Chaplin et al 2018

Figure S7: Venn diagram showing comparisons of our protein targets against targets published. **A -** a comparison between the core 112 common proteins targeted for glycation from this study with the specific AGE-modified protein targets from age-related (Bilova *et al*., 2017) and osmotic stress related studies (Paudel *et al*., 2016). **B –** a comparison between specific drought protein targets identified in this study with specific targets for osmotic stress (Paudel *et al*., 2016) and rosette age-related (Bilova *et al*., 2017). The Venn diagram was constructed using Venny 2.1.
